# Topological Heterogeneity of Protein Kinase C Modulators in Human T-Cells Resolved with In-Cell Dynamic Nuclear Polarization NMR Spectroscopy

**DOI:** 10.1021/jacs.4c05704

**Published:** 2024-09-25

**Authors:** Sarah A. Overall, Sina J. Hartmann, Quang H. Luu-Nguyen, Patrick Judge, Dorothea Pinotsi, Lea Marti, Snorri Th. Sigurdsson, Paul A. Wender, Alexander B. Barnes

**Affiliations:** † Institute of Molecular Physical Science, ETH Zurich, 8093 Zurich, Switzerland; ‡ Department of Chemistry, Stanford University, Stanford, California 94305-5080, United States; § Department of Biochemistry, Biophysics, & Structural Biology, Washington University in St. Louis, St. Louis, Missouri 63110, United States; ∥ Scientific Center for Optical and Electron Microscopy, ETH Zurich, 8093 Zurich, Switzerland; ⊥ Science Institute, University of Iceland, Dunhagi 5, 107 Reykjavik, Iceland

## Abstract

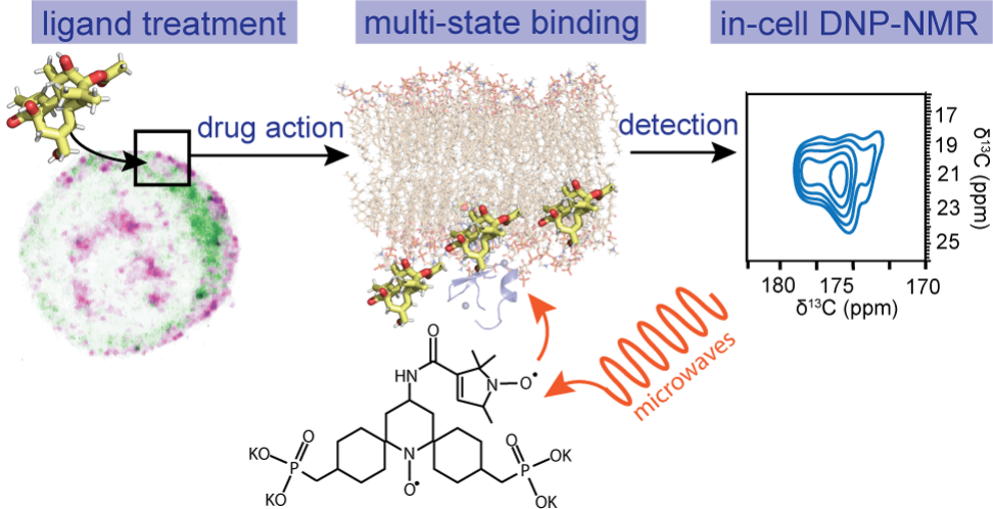

Phorbol ester analogs are a promising class of anticancer therapeutics and HIV latency reversing agents that interact with cellular membranes to recruit and activate protein kinase C (PKC) isoforms. However, it is unclear how these esters interact with membranes and how this might correlate with the biological activity of different phorbol ester analogs. Here, we have employed dynamic nuclear polarization (DNP) NMR to characterize phorbol esters in a native cellular context. The enhanced NMR sensitivity afforded by DNP and cryogenic operation reveals topological heterogeneity of ^13^C-21,22-phorbol-myristate-acetate (PMA) within T cells utilizing ^13^C–^13^C correlation and double quantum filtered NMR spectroscopy. We demonstrate the detection of therapeutically relevant amounts of PMA in T cells down to an upper limit of ∼60.0 pmol per million cells and identify PMA to be primarily localized in cellular membranes. Furthermore, we observe distinct ^13^C-21,22-PMA chemical shifts under DNP conditions in cells compared to model membrane samples and homogenized cell membranes, that cannot be accounted for by differences in conformation. We provide evidence for distinct membrane topologies of ^13^C-21,22-PMA in cell membranes that are consistent with shallow binding modes. This is the first of its kind in-cell DNP characterization of small molecules dissolved in the membranes of living cells, establishing in-cell DNP-NMR as an important method for the characterization of drug-membrane interactions within the context of the complex heterogeneous environment of intact cellular membranes. This work sets the stage for the identification of the in-cell structural interactions that govern the biological activity of phorbol esters.

## Introduction

**Figure 1:**
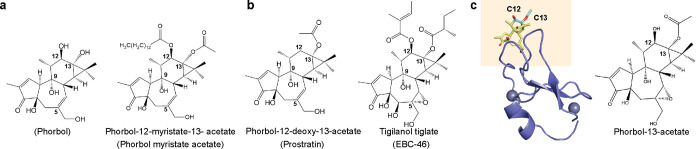
Chemical structures of tigliane and family members with different biological activities. (a) Structures of tumorigenic and inflammatory tiglianes which include phorbol, and phorbol myristate acetate. (b) Structures of antitumorigenic and anti-inflammatory tiglianes which include prostratin and tigilanol tiglate (EBC-46). (c) Crystal structure of phorbol-13-acetate bound to C1B domain of PKC-δ (PKC-δ-C1B) (pdb: 1PTR). The C12 and C13 moieties are shown in cyan. The structure of phorbol-13-acetate is shown on the right. The probable positioning of the membrane with respect to the complex is shown by the shaded area.

Phorbol esters are a group of tigliane diterpenoid compounds that have long been studied for their potent biological activities arising from binding to the C1 domain of protein kinase C (PKC) isoforms and other typical-C1 domain containing proteins.^1−4^ Phorbol esters with PKC-mediated biological activities are of high clinical relevance as antigen-enhanced cancer treatments,^5,6^ in HIV eradication,^7−9^ and wound healing.^10,11^ The C12 and C13 substituents (Figure 1) strongly influence the biological activity of phorbol esters. Phorbol-12-myristate-13-acetate (PMA) (Figure 1a), a tumor promoting competitor of the natural ligand of PKC-isoforms, diacylglycerol (DAG), exhibits low nM binding affinity.^12−15^ In contrast, prostratin (C12-deoxy-C13-acetate) and tigilanol tiglate (C6,C7-epoxy-12-tiglic-13-(*S*)-2-methylbutanoate family member) (Figure 1b), display HIV latency reversal and antitumor activity, while also maintaining low nanomolar binding affinity to PKCs.^16,17^ Variation of the C12 and C13 ester moieties of phorbol and more generally of tigliane diterpenes, modulates their activity toward PKC translocation to the cell membrane in various cell lines.^15,18^ The X-ray crystal structure of the phorbol derivative, phorbol-13-acetate bound to PKC demonstrates that the C12 and C13 groups do not directly contact PKC, but instead face away from the protein^19^ and probably orient toward the interior of the membrane (Figure 1c) as indicated by molecular dynamics (MD) simulation and a recent X-ray structure containing lipid molecules.^20−23^ Thus, the direct interaction of the lipid portion of these drug candidates with membrane bilayers likely contributes to the activity of the bound complex. Indeed, phorbol derivatives lacking lipophilic moieties at the C12 and C13 positions typically only promote weak or no membrane association of PKCs.^14,16^ The membrane interactions of phorbol esters have only been described indirectly by monitoring membrane binding of PKC which only distinguishes activators from nonactivators and does not distinguish inflammatory from noninflammatory activators, offering limited insight into the localization of the ligand. The structural interactions of the C12 and C13 groups which modulate phorbol activity remain wholly undetermined experimentally.

We recently reported a molecular dynamics study which predicts that the C1B domain of PKC-δ (PKC-δC1B) engages in cholesterol interactions when bound to phorbol-13-acetate (P13Ac), an interaction governed by the insertion depth (topology) of the complex.^22^ Furthermore, Ryckbosch and co-workers also reported different PKC:ligand topologies with different ligands over 500 μs molecular dynamics simulations.^23^ Deeper topologies were observed with inflammatory modulators. The topology therefore may be determined by the bound ligand. We hypothesize that the insertion depth determines the interaction with cholesterol and influences PKC-δ partitioning into lipid rafts, providing a compelling explanation for the disparate cellular activity of different PKC modulating drugs.^24^ The membrane positioning (topology) of the PKC-ligand complex may therefore selectively influence access of client proteins to the complex and thus downstream signaling. Uncovering the role played by the C13 and C12 groups in membrane topology would provide a structural and biophysical basis for the design of more efficacious, and specific PKC modulators. How the ligand might influence membrane topology of the complex is unknown largely due to a lack of experimental techniques which can extract information about the interactions between phorbol esters and lipid bilayers, particularly in the complex environment of cellular membranes.

Solid-state NMR methods are ideally suited to identifying and characterizing small molecule interactions with lipid bilayers at atomic resolution.^25^ However, the complexity of cellular membranes, particularly in composition, and microdomain architecture are impossible to recapitulate *in vitro* systems.^26^ Given the importance of membrane microdomains as signaling hubs, and as spatial regulators of protein–protein interactions,^27,28^ the need for the development of *in situ* methodologies for assessing membrane-drug interactions is pressing. Furthermore, the establishment of such methods will have extensive applications to *in situ* structural studies of membrane proteins, which account for over 20% of the human proteome, the potential impact of these methods is therefore clear.^29^ In-cell dynamic nuclear polarization NMR (DNP-NMR) is a powerful technique to address this need with the potential to determine structural interactions of small drug molecules dissolved in the complex environment of intact cellular membranes. In-cell DNP-NMR utilizes the large magnetic polarization of free electrons to transfer polarization to nuclei of interest.^30−32^ This technique yields 20–200 times increased NMR sensitivity allowing biological systems to be studied closer to their endogenous concentrations. Of further importance, the low temperatures of DNP-NMR (∼100 K) cryogenically preserve cellular samples allowing extended periods of magic angle spinning (MAS) for increased resolution with minimal cell perturbations^33,34^ and have demonstrated potential for structural studies in mammalian cells.^35^ However, at low temperatures, dynamic conformational sampling is suspended, resulting in inhomogeneous broadening by chemical shift heterogeneity and subsequent degradation of spectral resolution. The reduced resolution significantly limits the widespread application of DNP-NMR to uniformly labeled biomolecular systems. Additionally, potential changes in protein conformation and membrane structure upon freezing necessitates careful consideration of sample preparation conditions.

Here we demonstrate the power of in-cell DNP-NMR to detect membrane-dissolved site-specific ^13^C-labeled phorbol esters within intact JLat 9.2 T cells. To the best of our knowledge this is the first characterization of a membrane-bound small molecule within a living cell. We demonstrate sufficient resolution to identify distinct topologies within cells laying the groundwork for the utilization of in-cell DNP-NMR as the experimental technique of choice for the identification of ligand membrane topology in intact cell membranes and the characterization of drug-lipid interactions at the atomic scale.

## Results

### Strategic Design of ^13^C Probes for Phorbol Ester-Membrane Interaction Studies within Cells

**Figure 2:**
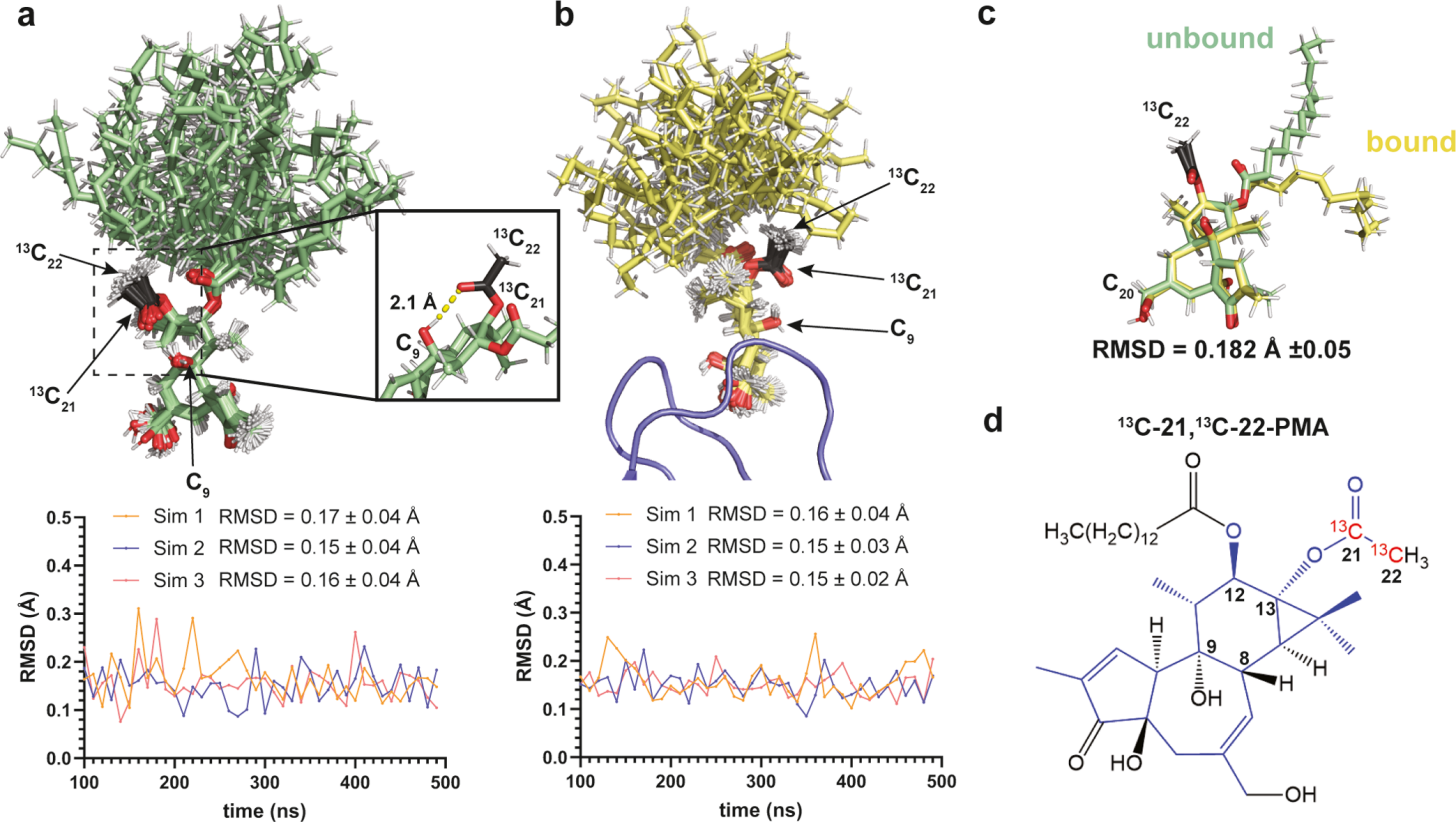
Conformational flexibility of the phorbol-12-myristate-13-acetate determined by MD simulation. (a) Conformational rigidity of unbound PMA in heterogeneous membranes. Superposition of 40 PMA conformations sampled every 10 ns over 400 ns, representative of three independent simulations in heterogeneous lipid bilayers which mimic the composition of the plasma membrane (membrane composition is given in SI-2). The position of ^13^C isotope labels is indicated on the structure in black. The inset highlights the hydrogen bond between the acetate C21 carbonyl oxygen and the C9 hydroxyl. (b) Conformational rigidity of protein bound PMA in heterogeneous membranes. Superposition of 40 structures of PMA bound to PKC-δC1B sampled every 10 ns over 400 ns simulation time. The RMSDs shown in (a, b) are calculated against the core pharmacophore atoms (shown in blue in (d)) of the first frame of the simulation and plotted as a function of time for each simulation. (c) Overlay of unbound and PKC-δC1B-bound PMA structures displaying a core pharmacophore RMSD of 0.18 Å. The average RMSD was calculated for 16 unbound PMA structures extracted every 25 ns from the simulation shown in (a) using the bound structure as reference for the RMSD calculation. RMSDs were then averaged from 10 separate calculations with 10 different bound reference structures spanning the full conformational space of the core pharmacophore (b). (d) Chemical structure of ^13^C-21,^13^C-22-PMA. The position of ^13^C isotope labels is indicated in red and the core pharmacophore atoms used for RMSD calculations are shown in blue.

To probe the interactions between phorbols and intact cell membranes with DNP NMR, we used an isotopically labeled phorbol-12-myristate-13-acetate (PMA) (Figure 1a) because of its high affinity and highly characterized biological activity. Before selecting the atoms for isotope labeling, we performed MD simulations of PMA in heterogeneous membranes mimicking mammalian plasma membrane composition^26^ in order to assess the potential contribution of conformational sampling of the phorbol pharmacophore to chemical shift heterogeneity, as conformational heterogeneity will diminish the chemical shift as a reporter of membrane environment or topology. We found the phorbol core pharmacophore to be conformationally rigid, with small variations in structure as evidenced by the average root mean square deviation (RMSD) of 0.16 ± 0.04 Å over 400 ns of simulation time. Interestingly, rotation of the C13 moiety was restricted due to hydrogen bonding of the acetate to the C9-hydroxyl group. As expected, the myristate moiety was conformationally flexible and so was not further considered for isotope labeling. Simulation of PMA bound to the C1B domain of PKC-δ also showed conformational rigidity with an average RMSD of 0.15 ± 0.03 Å (Figure 2b). Comparison of the bound and unbound structures revealed similar conformations with an RMSD of 0.18 ± 0.05 Å (Figure 2c), giving us a wide range of conformationally restricted atoms to choose from for isotope labeling. For ease of labeling, we selected both carbons of the acetate moiety, C21 and C22, (Figure 2d) as the groups bonded to the C13 atom of the phorbol scaffold have been found to be important in distinguishing tumor-promoting from nonpromoting phorbol esters.^18^ Labeling of two directly bonded ^13^C atoms also enables double quantum filtered NMR techniques for detection of small molecule ligands above natural abundance ^13^C atoms within cells.

### ^13^C-21,^13^C-22-PMA Localizes Exclusively to Cell Membranes

**Figure 3:**
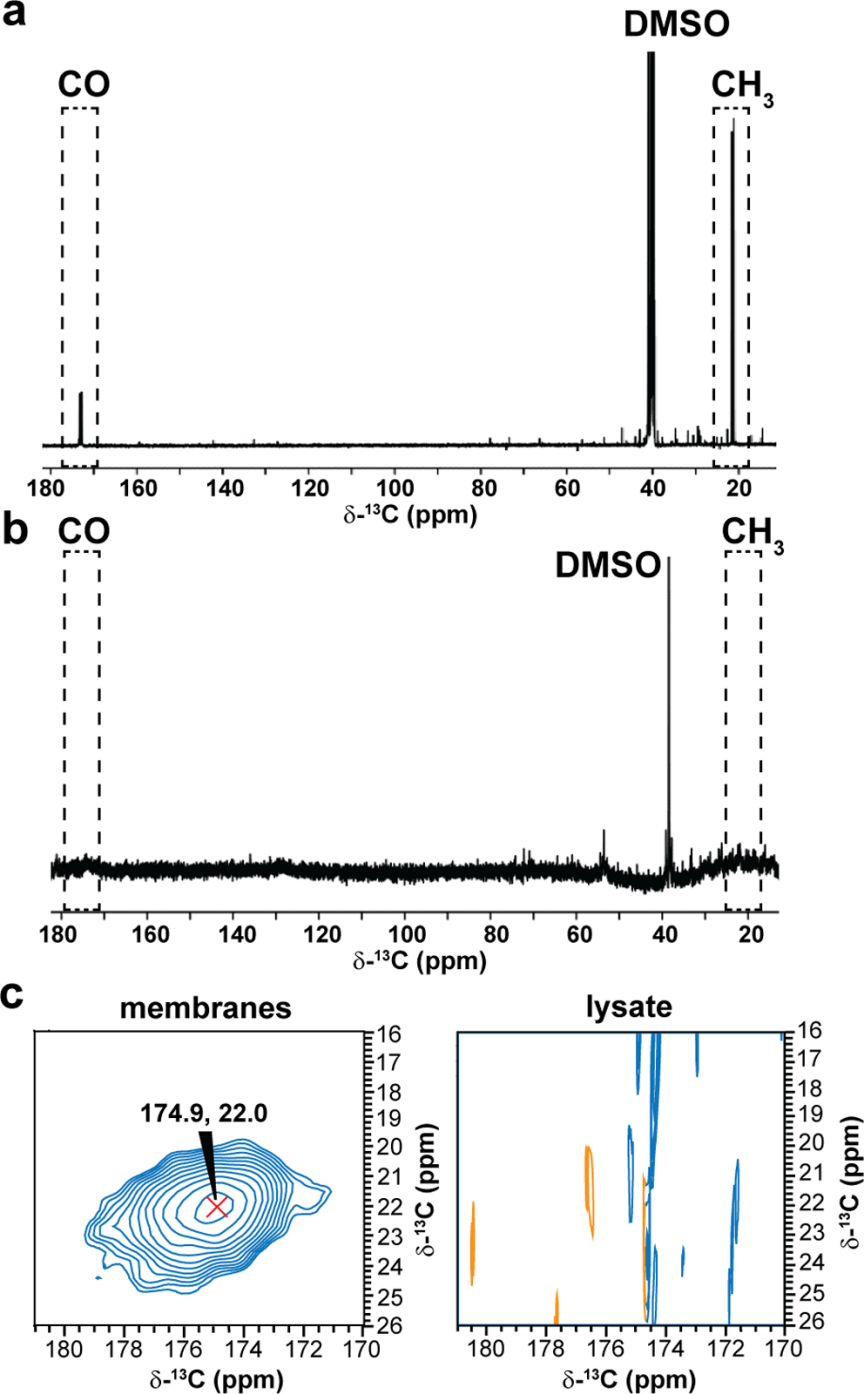
^13^C-21,^13^C-22-PMA association with cellular membranes. (a) ^13^C spectrum of 200.0 μM ^13^C-21,^13^C-22-PMA in DMSO showing the two J-coupled carbons (indicated by the dashed boxes) at 298 K. (b) ^13^C spectrum of 200 million JLat 9.2 T cells at 278 K treated with 200.0 μM ^13^C-21,^13^C-22-PMA. The regions where ^13^C-21,^13^C-22-PMA peaks are expected are indicated by the dashed boxes. Spectra were acquired at 1.2 GHz ^1^H Larmor frequency. (c) ^13^C–^13^C-DARR DNP spectrum of the membrane fraction (40 transients) (left) and soluble fraction (512 transients) (right) of lysed JLat 9.2 T cells from (b) prepared with 10% DMSO and 5.0 mM AsymPol-POK at 114 K. Solid-state spectra were acquired at 9 kHz MAS and 600 MHz ^1^H Larmor frequency.

^13^C-21,^13^C-22-PMA was synthesized from phorbol in three steps as described in SI-3. Characterization of ^13^C-21,^13^C-22-PMA in DMSO by solution-state NMR shows clear ^13^C signals diagnostic for the labeled C21 carbonyl and C22 methyl (Figure 3a). The concentration of undiluted PMA was confirmed to be 20.0 mM by NMR (SI-4). To characterize the chemical shifts associated with solvated ligand in cells we added ^13^C-21,^13^C-22-PMA to a final concentration of 200.0 μM to 200 million JLat 9.2 T cells and analyzed the sample by solution-state NMR at 28.1 T using a high signal-to-noise cryoprobe platform. No ^13^C signals corresponding to the carbonyl or methyl of ^13^C-21,^13^C-22-PMA could be detected (Figure 3b). Even low intensity ^13^C signals displayed no J-couplings, indicative of natural abundance isotopes suggesting that all the added ^13^C-21,^13^C-22-PMA was bound to membranes or large protein complexes and is therefore not detectable by solution-state NMR methods. To verify this, we lysed the same cells by sonication and separated the membrane fraction from the soluble fraction by ultracentrifugation. We performed DNP-NMR on each of these fractions and only detected cross peaks between the carbonyl and methyl group of ^13^C-21,^13^C-22-PMA in the membrane fraction with a signal-to-noise ratio of 38.8 (Figure 3c), indicating that all ^13^C-21,^13^C-22-PMA was localized to membranes. Even with more than an order of magnitude more transients collected on the lysate fraction, no cross peaks were observable. It is unlikely that the lack of ligand detection in the lysate fraction is due to increased line-broadening of solvated ligand, a phenomenon observed in protein spectra at cryogenic temperatures linked to conformational sampling of water exposed residues.^36^ The lack of conformational sampling of the labeled ligand together with the increased number of scans used to collect the lysate spectrum indicates that this kind of broadening does not account for the lack of ligand detection in the soluble fraction, however, we cannot rule out the possibility.

### Detection of ^13^C-21,^13^C-22-PMA in Cell Membranes

**Figure 4:**
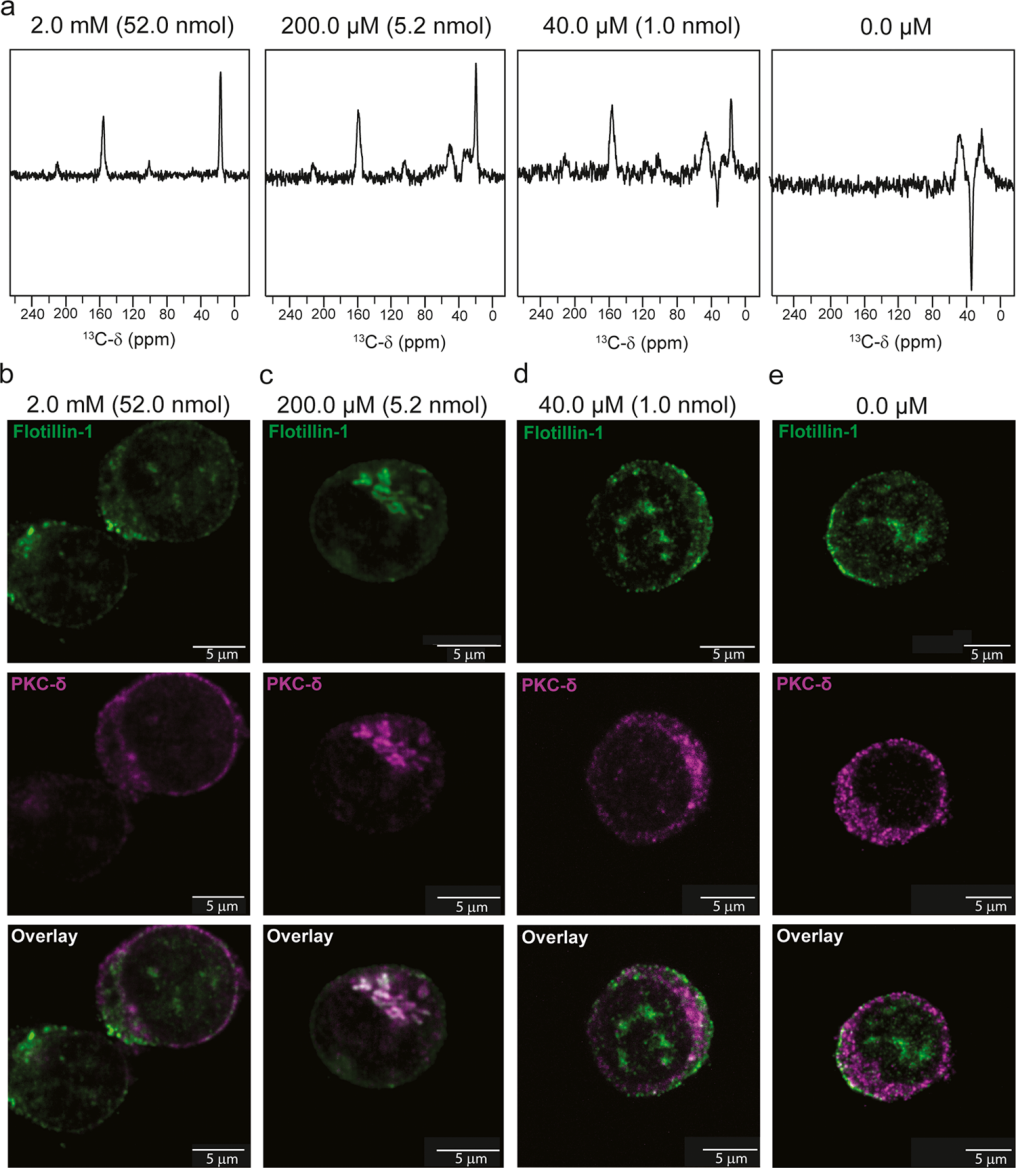
Detection limit of ^13^C-21,^13^C-22-PMA and PKC-δ localization in cells. (a) Titration of ^13^C-21,^13^C-22-PMA in JLat 9.2 T cells analyzed by DNP-NMR. ^13^C SPC5 double quantum spectra of JLat 9.2 T cells treated with ^13^C-21,^13^C-22-PMA at the concentrations indicated. All spectra were acquired at 600 MHz ^1^H Larmor frequency, 9 kHz MAS and 114 K stator temperature with 1024 (2.0 mM), 10,240 (200.0 μM), 20,560 (40.0 μM) and 40,960 (0.0 μM) transients collected, respectively. Values given in brackets indicate the total number of PMA molecules exxpected per rotor (b–e) Confocal microscopy of JLat 9.2 T cells treated with ^13^C-21,^13^C-22-PMA. JLat 9.2 T cells prepared for DNP in (a) (5 min incubation with ^13^C-21,^13^C-22-PMA) were fixed with 4% paraformaldehyde and stained with anti-PKC-δ (magenta) and antiflotillin-1 (green) antibodies and AlexaFluor-647 and CF568 secondary antibodies, respectively. Colocalization is indicated by white staining in the overlay panels. Cells were imaged using a Nikon Ti2 microscope equipped with a confocal rescan module. The images shown are representative slices through the cell, taken from a three-dimensional *z*-stack (except for (e) which is a single plane), the full image series is shown in SI-8.

After establishing that ^13^C-21,^13^C-22-PMA localizes to cellular membranes, we investigated the detection limit of ^13^C labeled molecules in intact cells by DNP-NMR. Natural abundance ^13^C atoms in cell samples are readily detected using DNP due to the significant gains in sensitivity. We obtained cellular enhancements of 20–45  (SI-5) at 5.0 mM concentration of the known polarizing agent AsymPol-POK (structure is shown in SI-6),^37^ at 600 MHz ^1^H Larmor frequency. Double quantum filtering (DQ-filter) was used to suppress natural abundance ^13^C signals and specifically visualize the labeled ligand in one-dimensional (1D) spectra. Utilizing the SPC5 DQ-filter sequence,^38^ we titrated ^13^C-21,^13^C-22-PMA down to 40.0 μM (1.6–1.0 nmol/rotor) where we could still readily detect ^13^C labeled carbonyl and methyl resonances from the acetyl group of ^13^C-21,^13^C-22-PMA (Figure 4a). This equates to an upper limit of ∼60.0–40.0 pmol ^13^C-21,^13^C-22-PMA per million cells (1.6–1.0 nmol total ^13^C-21,^13^C-22-PMA) which is orders of magnitude below the biologically active concentration typically used in cell assays (20–0.5 nmol per million cells) as calculated from 1 μM to 100 nM PMA in 10 mL of media with 2–0.5 × 10^6^ cells per 10 mL.^39^ The efficiency of SPC5 is typically low, having a theoretical maximum transfer efficiency of 50%.^38^ We observed a range of transfer efficiencies from 12 to 36% in a model sample of U–^13^C-alanine mixed with natural abundance glycine which was dependent on the resonance frequency offset (SI-7). However, the transfer efficiency of SPC5 typically reported in the literature for biomolecules is around 8–20%,^40,41^ therefore we expect our transfer efficiencies in cells to be within a similar range. However, we were unable to determine the transfer efficiency in cell samples as we cannot observe the ^13^C-21,^13^C-22-PMA labels without the DQ-filter due to the large enhancement of natural abundance ^13^C in 1D CP spectra masking the signals from PMA. Nevertheless, even with the low efficiency of the DQ-filter compared to cross-polarization and multiple contact sequences, we are able to detect ^13^C-labeled small molecules at a similar order of magnitude as that achieved in other studies using labeled rare-nuclei within cells.^42^ Our results clearly demonstrate the feasibility of utilizing ^13^C labels for in-cell drug studies by DNP-NMR.

The translocation of PKC-δ to the plasma membrane and other membrane structures within cells is the gold standard for measuring the activity of PKC modulators.^43,44^ PKC-δ is also known to localize to other subcellular compartments in response to PKC modulators, which influences the biological outcome of PKC-δ activation.^45^ We therefore imaged ^13^C-21,^13^C-22-PMA stimulated JLat 9.2 T cells for PKC-δ localization by confocal microscopy to demonstrate the biological significance of the concentrations detected by DNP-NMR. We immediately fixed an aliquot of JLat 9.2 T cells prepared for DNP prior to flash freezing for confocal imaging. At 2.0 mM ^13^C-21,^13^C-22-PMA, clear plasma membrane recruitment of PKC-δ was observed with strong fluorescence detection of PKC-δ at the periphery of the cell (Figure 4b). At 200.0 μM ^13^C-21,^13^C-22-PMA we observed a golgi staining pattern, which colocalized intracellularly with flotillin-1 (Figure 4c), a membrane protein typically associated with lipid rafts at the plasma membrane, indicating internalization from the plasma membrane. Golgi translocation of PKC-δ has been reported in response to ceramide-based modulators^46,47^ as well as the phorbol ester PDBu^48^ which is C1B domain dependent. Golgi localization is generally associated with apoptosis in HeLa and CHO cells.^49^ However, PMA induced golgi translocation of PKC-δ has not been reported in Jurkat T cell lines. Furthermore, the time scale for flash freezing after the addition of PMA is relatively short it is within the well characterized range of PKC-δ recruitment times of 2–60 min typically observed with 1 μM to 100 nM-PMA.^43,47,50^ However, the precise kinetics of recruitment is dose dependent and cell type dependent. Thus, at 2.0 mM (2000 pmol/million cells) and 200.0 μM (200 pmol/million cells) the topological landscape of ^13^C-21,^13^C-22-PMA will contain protein bound states in association with membrane structures. Below 200.0 μM we observed no changes in the localization of PKC-δ (Figure 4d,e) suggesting that at 40.0 μM (40 pmol/million cells) few or no protein bound states contribute to the ^13^C-21,^13^C-22-PMA topological landscape at the time of flash freezing. However, we expect the concentration of typical-C1 domain containing proteins in cells to be ∼0.16 pmol per million cells^1^ (≈0.003 nmol/rotor), which is below our estimated detection limit. Thus, it is unlikely that protein bound states are explicitly visible in the spectra.

### Chemical Shift Distribution of ^13^C-21,^13^C-22-PMA in Cell Membranes

**Figure 5:**
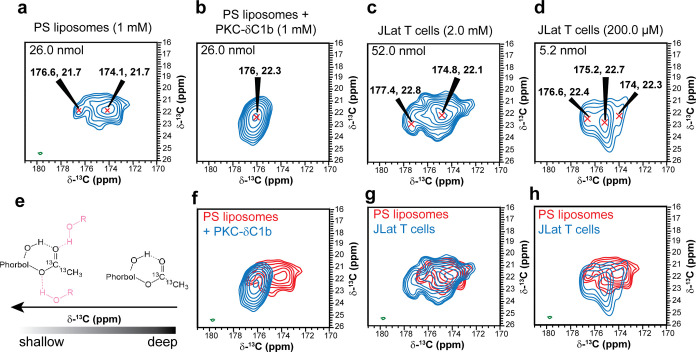
Topological heterogeneity of ^13^C-21,^13^C-22-PMA in cells and model membranes. 2D-^13^C–^13^C DARR correlation spectra of ^13^C-21,^13^C-22-PMA in *in vitro* and *in vivo* systems at the concentrations indicated. Total amounts of ^13^C-21,^13^C-22-PMA expected per sample are given inside each plot. (a) Spectra of ^13^C-21,^13^C-22-PMA in PS liposomes. (b) Spectra of ^13^C-21,^13^C-22-PMA bound to PKC-δ-C1B in PS liposomes. (c) Spectra of 2 mM ^13^C-21,^13^C-22-PMA in JLat 9.2 T cells. (d) Spectra of 200 μM ^13^C-21,^13^C-22-PMA in JLat 9.2 T cells. Data was collected at 600 MHz ^1^H Larmor frequency at 9 kHz MAS, 114–118 K stator temperature. All experiments were acquired with 20 ms DARR mixing, 512 transients and 600 increments in the indirect dimension. The observed chemical shifts are indicated and are internally referenced to DMSO. All samples were cryopreserved with 10% DMSO and prepared with 5.0 mM AsymPol-POK as the polarizing agent. (e) Proposed source of ^13^C chemical shift heterogeneity as a function of membrane depth. (f–h) Overlay of spectra from PS liposomes (red) with (f) PS liposomes+ PKC-δC1b, (g) JLat T cells (2 mM) and (h) JLat T cells (200 μM).

The topological states occupied by ^13^C-21,^13^C-22-PMA can be defined in terms of membrane environments associated with the insertion depth and thus accessibility to polar groups. To characterize the topological states of ^13^C-21,^13^C-22-PMA in JLat 9.2 T cells we utilized^13^C–^13^C DARR^51^ correlation spectroscopy to compare the chemical shift distribution of ^13^C-21,^13^C-22-PMA in cells and in model membrane systems. We used phosphatidylserine (PS) liposomes as our *in vitro* model membrane system as PS is essential for membrane binding of PKC-ligand complexes. To characterize the potential for resolving topology information through the chemical shift we chose a simple membrane system of 100% PS to minimize chemical shift contributions from potential interactions with diverse lipid headgroup chemistries. We observed cross peaks between the two labeled carbons in ^13^C-21,^13^C-22-PMA in both cells and model membranes (SI-9 and Figure 5). In PS liposomes, we observed a dominant cross-peak at 174.1 and 21.7 ppm between the carbonyl and methyl resonance, respectively, as well as a minor cross-peak at 176.6 and 21.7 ppm (Figure 5a). In the presence of excess recombinant PKC-δ-C1B protein these resonances shifted to a single peak at 176.0 and 22.3 ppm (Figure 5b) with a line width at full width half height (fwhh) of 1.51 ppm (SI-10). These downfield-shifted resonances indicate a more electronically deshielded environment around ^13^C-21,^13^C-22-PMA when in close proximity to the polar backbone atoms of PKC-δ-C1B in PS liposomes. In JLat 9.2 T cells, at 2 mM ^13^C-21,^13^C-22-PMA, a similar chemical shift profile to that of PS liposomes was observed (Figure 5c,g), with a small shift downfield of the dominant peak to 174.8, 22.2 ppm. The similarity between the spectra obtained in PS liposomes and 2 mM JLat T cells indicates that the PMA chemical shift is relatively insensitive to lipid composition. Reducing the concentration of ^13^C-21,^13^C-22-PMA to 200.0 μM revealed three distinct carbonyl resonances at 176.6, 175.2, and 174.0 ppm (Figure 5d,h) that were further shifted downfield compared to 2 mM JLat 9.2 T cells. We attribute these large chemical shift changes to the hydrogen bonded states of the ^13^C-carbonyl (Figure 5e), with increased hydrogen bonding of the flanking oxygens giving rise to larger downfield chemical shift changes of the acetate carbonyl.

**Figure 6:**
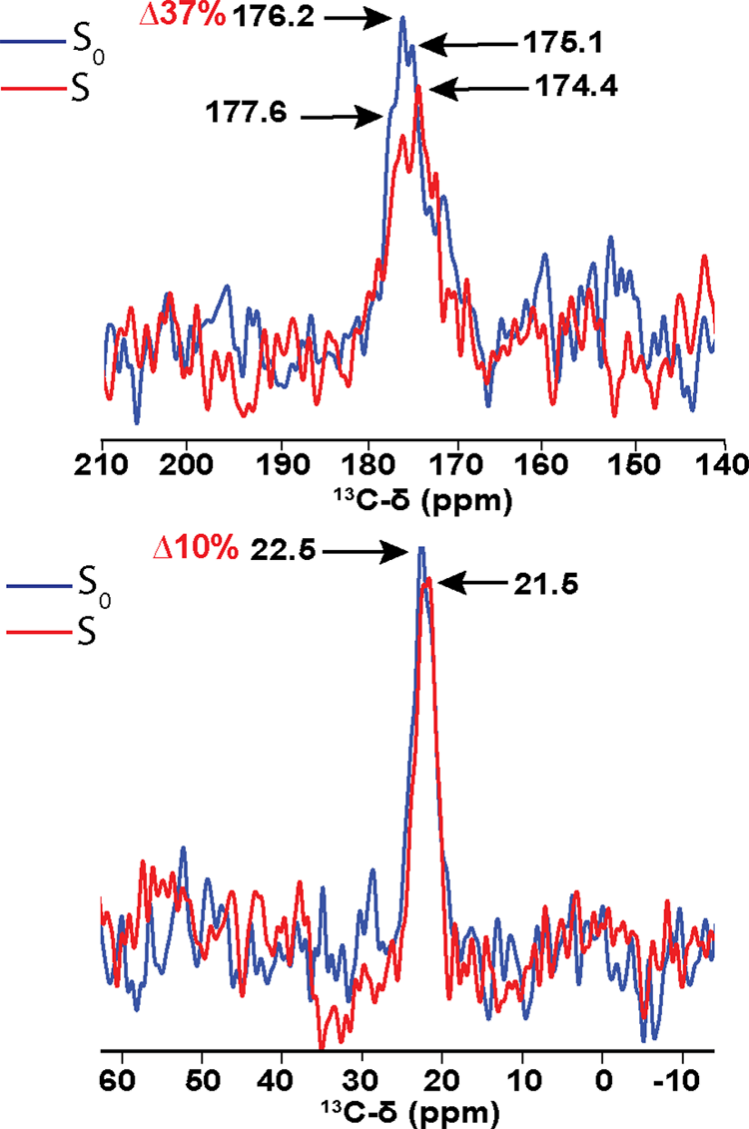
DQF-^31^P dephased REDOR of 200 μM ^13^C-21,^13^C-22-PMA in JLat 9.2 T cells. Top panel shows the ^13^C carbonyl of PMA while the bottom panel shows the ^13^C methyl of PMA. Spectra were acquired interleaved with an SPC5 double quantum filter at 9 kHz MAS and 4.45 ms dephasing time. Reference (S_0_) experiment is shown in blue and the dephased (S) experiment is shown in red. Percentages in red indicate the difference in peak intensities between S_0_ and S spectra for the indicated peak.

It is expected that shallow binding topologies will be characterized by closer proximity of the PMA ^13^C labels to the phosphate headgroup of membrane lipids. To verify that these peaks are associated with shallow binding topologies we performed ^31^P dephased REDOR on 200 μM JLat 9.2 T cells, in which we expect the shallowest topologies based on the ^13^C-carbonyl chemical shifts. In order to visualize the PMA ^13^C labels, we added an SPC5 double quantum filter to the REDOR experiment. At 4.45 ms dephasing time, we observe reduction in the intensity of the both the carbonyl and methyl labels of PMA (Figure 6), which was more pronounced on the downfield peaks carbonyl peaks indicating that the observed downfield shifted peaks are in closer proximity to phospholipid headgroups.

## Discussion

A major challenge of understanding how phorbol esters regulate PKCs is the detection of small molecule interactions with cellular lipids and other membrane components. The C12 and C13 moieties which face the membrane environment when bound to PKC appear to play a key role in determining the biological activity of phorbol esters.^14,43^ The disparity between the *in vitro* biochemical activity measured for some tigliane analogs and their *in vivo* activity highlights the importance of characterizing and understanding these interactions within the complex structure and composition of cellular membranes of intact cells.^18^ Solid-state NMR has long been used to study the interaction of small molecule drugs and peptides with model membrane systems.^52−56^ In recent years, solid-state methods have been applied to whole bacterial systems often lyophilized^57,58^ and membrane vesicles prepared from cells for drug-membrane studies at ambient temperatures.^59,60^ However, these systems lack the structural complexity of cell membranes. Here we demonstrate the feasibility of using ^13^C NMR spins as reporters of small molecule membrane insertion depth and environment (topology) in cell membranes of intact mammalian cells. The two pillars of this strategy are DNP as well as the combination of ^13^C-labeling of adjacent carbon atoms and double quantum filtering techniques. This approach allows the detection of therapeutically relevant quantities of ligands within cellular membranes together with topological information on the surrounding membrane environment. With this method, we demonstrate for the first time that membrane association of PMA in cells is a multistate phenomenon.

The limit of detection is dependent on many factors, such as the DNP enhancement, the number of cells (cell size and therefore stage in the cell cycle), the gyromagnetic ratio of the detection nucleus, the efficiency of magnetic polarization transfer schemes and the sample temperature. In this work we chose to use JLat 9.2 T cells, a Jurkat T cell line, which contains the HIV genome integrated into the JLat 9.2 T cells genome.^61^ Reactivation of latent HIV in JLat 9.2 T cells by phorbol esters is dependent on PKC activation,^62^ a phenomenon we have previously reported to detect by ^15^N DNP-NMR.^63^ We are particularly interested in understanding the structural underpinnings of the potency of tigliane analogues in HIV latency reversal which is the key “kick” step in the “kick and kill” approach to an HIV cure.^9^ An additional advantage of studying JLat 9.2 T cells, is that nearly 10 times as many JLat 9.2 T cells can be packed into a 3.2 mm rotor compared to the more commonly used HEK cells. JLat 9.2 T cells have a smaller cytoplasmic volume compared to HEK cells, such that a larger proportion of the rotor volume contains lipid bilayers for dissolving PMA. This makes a significant contribution to our ability to detect ∼60.0–40.0 pmol of ^13^C-21,^13^C-22-PMA per million cells, which is comparable to the detection limits reported by others using more efficient transfer schemes and lower magnetic fields (and therefore larger enhancements).^42^ Importantly, we are able to detect physiologically relevant quantities of ^13^C-21,^13^C-22-PMA typically used at 100 nM, equating to ∼100.0 pmol per million cells/mL. However, the characterization of membrane topologies at these concentrations with 2D correlation experiments will likely require improvements in enhancements using pulsed DNP, higher magnetic fields and sample temperatures approaching 4 K.

The chemical shift of the reporter nucleus directly correlates with its electronic environment surrounding the reporter nucleus. Increased hydrogen bonding and electronegativity result in downfield chemical shifts, while apolar environments result in upfield changes in the chemical shift. ^13^C-21,^13^C-22-PMA was expected to exist in a single conformation in both the protein bound and unbound states based on the MD simulations. As a result, we suggest that the multiple distinct chemical shifts observed by DNP-NMR are evidence for distinct hydrogen bonded states of the ^13^C-carbonyl due to topological heterogeneity of ^13^C-21,^13^C-22-PMA in cellular membranes. That increased hydrogen bonding occurs with shallower binding modes, rather than conformational heterogeneity of the labeled acetate as the acetate group is fixed in a single conformation due to hydrogen bonding with the C9 hydroxyl. The absolute value of the chemical shift of the PMA methyl may be influenced by the proximity of the hydrophobic myristate chain. However, such effects cannot explain the resolvable difference in the chemical shifts that we suggest is best explained by differences in hydrogen bonding. We define membrane topology to represent the position within the membrane occupied by ^13^C-21,^13^C-22-PMA, where deep topologies correlate with apolar environments and shallow topologies correlate with increased polarity of the environment. At the lowest concentration of ^13^C-21,^13^C-22-PMA analyzed by 2D spectroscopy, we observed the most deshielded chemical shifts, consistent with an increased polarity of the local environment. This is further supported by the pronounced ^31^P REDOR dephasing observed on the most downfield shifted carbonyl peaks. The reduced dephasing effect on the methyl of PMA could be due to longer distances between the PMA methyl and phosphorus atom of the lipid headgroups, but residual methyl rotation could also reduce the dipolar coupling, giving an under-estimation of the dipolar coupling. We interpret this data to indicate that ^13^C-21,^13^C-22-PMA prefers a shallow membrane topology, with increased interactions with lipid headgroups and water. At large excesses of ^13^C-21,^13^C-22-PMA, deeper topologies dominate the spectrum. Alternatively, at these high concentrations, nonspecific binding to other proteins, or potentially C1A domains might occur as has been shown for isolated C1A domains. Whether such binding is distinguishable by NMR remains to be determined. Of note, these resolvable environments seen in intact cells were largely lost upon sonication and fractionation of JLat 9.2 T cell membranes consistent with the resolvable heterogeneity being directly related to actively maintained membrane structures. This highlights the need for structural techniques compatible with intact cells which have distinct lipid compositions of the inner and outer leaflets and of lipid rafts.^64^

The development of methodologies for determining drug-membrane topology of membrane active drugs within the cellular context is critical for furthering our understanding of how phorbols and other membrane associated drugs enact their cellular activities. Importantly, the methodology presented here is directly translatable to protein–membrane studies and offers a path for directly accessing structural information about protein–lipid interactions within cellular membranes, relevant to over 20% of the human proteome.^29^

Localization of PKC to membrane microdomains influences the protein substrates phosphorylated by PKC, which determines subsequent transcriptional activation and therefore, biological outcomes. Temporally modulated localization could similarly impact PKC signaling outcomes and thus a preferred topology could significantly shift the kinetics of PKC recruitment to lipid rafts toward inflammatory outcomes. Our data suggests that a shallower topology is the preferred membrane interaction mode of PMA in cellular membranes. The establishment of a causal link between membrane topology and cellular outcomes might be established using the methodology outlined in this work through a systematic comparison of phorbol analogs with distinct biological effects.

## Conclusions

We have revealed the potential for in-cell DNP NMR spectroscopy to provide topological information about membrane bound small molecules within cells. Solid-state DNP offers sufficient resolution to distinguish multiple local environments surrounding ^13^C-labeled phorbols present in cellular membranes. No other technique allows for the gathering of topological information on small drug molecules in intact cell membranes at the Angstrom scale. Given the importance of membrane-drug interactions in the effects of many drugs, in-cell DNP will be an important method in the atomic characterization of small molecules in cell membranes. Expanding our understanding of how small molecules interact with specific membrane domains will open up the possibility of specifically targeting these structures in drug design principles^65^ with clear applicability to many drug membrane systems such as anesthetics and the nonselective effects of adrenergic receptor agonists and antibiotics.^52,65−67^ This work offers a pathway for characterizing how drugs interact with membrane microdomains in the complex and heterogeneous environment of intact cells, furthering our understanding of how these interactions influence drug efficacy.

## Supporting Information Available

Experimental materials and methods including lipid compositions for molecular dynamics simulations, synthetic methods for synthesis of ^13^C-21,^13^C-22-PMA including ^1^H and ^13^C NMR data of all compounds synthesized; ^13^C NMR spectra characterization of DNP experiments including enhancements, SPC-5 double quantum transfer efficiencies and the full 2D-DARR correlation spectrum; full series of confocal images collected as a *z*-stack from which the figure images are taken, and analysis of MD simulation equilibration and system stability (PDF)

## Abbreviations

PMA: phorbol myristate acetate
DNP: dynamic nuclear polarization
MAS: magic angle spinning
PKC: protein kinase C
MD: molecular dynamics
