# Supplementary material for: Topological Heterogeneity of Protein Kinase C Modulators in Human T-Cells Resolved with In-Cell Dynamic Nuclear Polarization NMR Spectroscopy
Source: J Am Chem Soc. 2024 Sep 25;146(40):27362–72. doi: 10.1021/jacs.4c05704 (PMC11468733; doi:10.1021/jacs.4c05704)
Supplement: Supplementary file 1 — ja4c05704_si_001.pdf [file ja4c05704_si_001.pdf]

Supporting Information

Topological heterogeneity of protein kinase C modulators in human T-cells resolved with in-cell dynamic nuclear polarization NMR spectroscopy

Sarah A. Overall[1*], Sina J. Hartmann[1], Quang H. Luu-Nguyen[2], Patrick Judge[3], Dorothea Pinotsi[4], Lea Marti[1], Snorri Th. Sigurdsson[5], Paul A. Wender[2] and Alexander B. Barnes[1*]

[1]Institute of Molecular Physical Science, ETH Zurich, 8093 Zurich, Switzerland
[2]Department of Chemistry, Stanford University, California, 94305-5080, USA
[3]Department of Biochemistry, Biophysics, & Structural Biology, Washington University in St. Louis, Missouri 63110, USA
[4]Scientific Center for Optical and Electron Microscopy, ETH Zurich, 8093 Zurich, Switzerland
[5]Science Institute, University of Iceland, Dunhagi 5, 107 Reykjavik, Iceland
*Correspondence: soverall@ethz.ch and abarnes@ethz.ch

Table of Contents

SI-1: Materials and Methods……………………………………………………………………2
SI-2: Membrane compositions used in MD simulations……………………………………….5
SI-3: Synthesis of ^{13}C-21,^{13}C-22-PMA…………………………………………………………6
SI-4: Measurement of ^{13}C-21,^{13}C-22-PMA concentration by NMR…………………………11
SI-5: DNP enhancements of JLat 9.2 T cells with 5 mM AsymPolPOK………………………12
SI-6: Structure of AsymPolPOK……………………………………………………………12
SI-7: Efficiency of SPC5 double quantum filter with U-^{13}C-Alanine…………………………..13
SI-8: Image series of PKC-δ translocation with ^{13}C-21,^{13}C-22-PMA…………………………14
SI-9: 2D-DARR spectrum of ^{13}C-21,^{13}C-22-PMA in JLat 9.2 T cells……………………….16
SI-10: Supporting Information 10: Linewidth analysis of DARR cross peaks…………….....17
SI-11: Minimization and equilibration of membrane systems in GROMACs……………..…18
References…………………………………………………………………………………19

Supporting Information 1: Materials and Methods

Synthesis of ^{13}C-21,^{13}C-22-PMA

See supplementary information (SI-3) for detailed procedure and spectroscopic characterization.

Cell Culture

JLat 9.2 T cells, a Jurkat T cell line containing genomically integrated HIV as previously described[1] were cultured in complete RPMI (2.0 mM L-Glutamine,10% v/v Fetal Bovine Serum (FBS) (Gibco), 100 U/ml penicillin-100 μg/ml streptomycin (Gibco) and 10 mM sodium pyruvate (Gibco) at 37 °C under a humidified 5% CO_2 atmosphere. Cells were counted using a hemocytometer and trypan blue (Sigma-Aldrich) staining. A 1:1 dilution of cell culture with 0.4% trypan blue was observed under a light microscope, blue cells were counted as dead and non-blue refractive cells were counted as viable.

Confocal Microscopy

JLat 9.2 T cells were prepared for DNP as described below. Prior to freezing, 5 μL of cell pellet was transferred to a microfuge tube and the cells were immediately fixed with 4% paraformaldehyde (PFA) overnight at 4 °C. The cells were washed three times with phosphate buffered saline (PBS) then stained with primary antibody (mouse IgG anti-human-PKC-δ (Sigma-Aldrich) (1/200 dilution) and rabbit IgG anti-human Flottilin-1 (Sigma-Aldrich) (1/250 dilution)) for 1 h at room temperature. The cells were washed three times with PBS then stained with secondary antibody (AlexaFluor-647 goat IgG anti-mouse-IgG (Invitrogen) (1/1000 dilution) and CF568 goat-IgG anti-rabbit-IgG (Thermofisher) (1/1000 dilution)) for 1 h at room temperature. The cells were washed 5 times with PBS then resuspended in 50 μL PBS, transferred to 8 well glass slides (Ibidi) and 300 μL of CyGel (Biostatus) was added to each well to immobilize the cells. Confocal microscopy was performed with a Nikon NSTORM (Nikon UK, Ltd) system equipped with a Re-scan Confocal Microscope RCM1 (Confocal.nl, Amsterdam, the Netherlands). We used an sCMOS camera (Orca Flash 4.0 V2) and a Nikon SR Apochromat TIRF objective 100x / 1.49 with oil immersion. The different laser excitations were at 561 nm, and 647 nm. The setup was fully controlled, and image acquisition was performed using the NIS-Elements software (Nikon). The implemented re-scan unit provides an enhancement in resolution from 240 nm to 170 nm. Images were processed in ImageJ where the brightness was adjusted such that the brightest pixels had a greyscale value of 50 and the contrast adjusted to make fluorescent structures distinguishable from non-fluorescent structures.

NMR Sample preparation

In-cell samples were prepared by taking 40×10^6 live JLat 9.2 T cells, washed with ice cold PBS, and pelleted (340 *g*, 5 min, 4°C). Cell viability was determined by trypan blue exclusion with light microscopy. Cell viability ranged from 95-97% viable cells. The cell pellet was resuspended in deuterated PBS and incubated on ice for 10 min to allow exchange of intracellular water with D_2O. After having pelleted the cells once more (340 *g*, 5 min, 4 °C) AsymPolPOK[2] (5.0 mM) and $^{13}C_{21,22}$-PMA were added to the cell pellet and resuspended giving a final DMSO concentration of 10% (v/v). The sample was incubated on ice for 5 min and pelleted (340 *g*, 1 min, 4 °C) into a sapphire rotor (Bruker Biospin) using custom made filling tools.[3] Cellular integrity post DNP analysis in our setup has been extensively characterized in a previous publication.[4]

PMA amounts were calculated using the following parameters: 26 million cells per rotor (1 million cells per μL as counted experimentally). 26 μL rotor volume. All the drug is assumed to bind to the cells and amounts per cell are calculated based on the 40 million cells prepared prior to packing. The values given therefore represent the highest possible amounts (i.e: 100% cell binding). The values given for therapeutic amounts were calculated based on typical assay parameters of: 1 mL of cell culture volume at 1 million cells per mL.

JLat 9.2 T cell membranes and lysates were prepared by pelleting 200 million ^{13}C-21,^{13}C-22-PMA treated JLat 9.2 T cells and removing all supernatant. The pellet was sonicated for 15 s five times with 1 min on ice in between to minimize heating. Complete cell destruction was confirmed by light microscopy. AsymPol-POK was added to the sonicated pellet to a final concentration of 5.0 mM along with 10% DMSO and transferred to a sapphire DNP rotor with custom made filling tools and centrifuged for 6 h at 160,000 g in an ultracentrifuge. All the supernatant was removed and centrifuged into a second sapphire rotor to produce the lysate sample.

Solid-State DNP-NMR

Samples were measured on a 14 T Bruker DNP spectrometer operating at 600 MHz ^{1}H Larmor frequency, the spectrometer was equipped with a harmonic 365 GHz gyrotron as well as a 3.2 mm HX LTMAS probe. The samples were spun at 9 kHz with a sample temperature of 104-108 K without microwaves and 114–118 K with microwaves. 1D-^{13}C spectra were acquired using a cross polarization (CP) scheme with ^{1}H spin-locking amplitude of 70 kHz over a 1.5 ms linear ramp centered at 60 kHz and ^{13}C spin locking amplitude of 50 kHz. Data was acquired under spinal64 ^{1}H decoupling at 112 kHz with 512 transients and recycle delay of 1.26 x T_1 (2 s).

Double quantum filtering was achieved using the SPC5 recoupling scheme[5] with 45 kHz ^{13}C recoupling radio-frequency amplitude over 555 μs excitation and 555 μs reconversion time with 100 kHz continuous wave ^{1}H decoupling with Lee-Goldberg offset and a ^{13}C carrier frequency centered at 170 ppm.

2D DARR experiments were carried out using the same parameters as described for CP experiments with 20 ms recoupling with 9 kHz ^{1}H irradiation. 2D data was acquired with 2048 points in the direct dimension and 600 points in the indirect dimension and 32 transients.

DQF-REDOR data were collected by placing an SPC5 double quantum excitation and reconversion block before the REDOR dephasing block using the same parameters optimized for the SPC5 experiment. REDOR was performed with 7.1 μs π dephasing pulses on ^{31}P and 9 μs π refocusing pulse on ^{13}C under continuous wave ^{1}H decoupling at 102 kHz followed by ^{13}C detection under spinal64 decoupling at 112 kHz. 1D data was analyzed in CcpNmr Analysis.v3,[6] 2D data was analyzed with NMRFAM sparky[7] all accessed through NMRBox.[8] All spectra are processed with 50 Hz line broadening and 512 points. Line shapes were fit using DMFit software[9].

Solution-State NMR

^{13}C-21,^{13}C-22-PMA in DMSO was diluted in d_6-DMSO to 200.0 μM, placed in a 5 mm NMR tube and direct ^{13}C spectrum acquired using a 45° flip angle pulse acquire experiment with Waltz16 ^{1}H decoupling at 500 MHz ^{1}H Larmor frequency at 298 K.

Measurement of ^{13}C-21,^{13}C-22-PMA concentration was done by preparing ^{13}C-21,^{13}C-22-PMA in d_6-DMSO as a 1 in 10 dilution from the stock solution (SI-4). Natural abundance anisole was added to the solution to give a 4 mM final concentration from a neat stock solution as the standard compound. 1D ^{1}H spectra were acquired using a 35° flip angle pulse acquire experiment with the recycle delay set to 6 x T_1 of the longest relaxing component as measured using an inversion recovery experiment. All experiments were acquired on a 700 MHz ^{1}H Larmor frequency spectrometer at 298 K.

In-cell samples were prepared by taking 200 million JLat 9.2 T cells, washing with PBS then adding ^{13}C-21,^{13}C-22-PMA in DMSO to 200.0 μM. The cells were centrifuged into a 3 mm NMR tube and the excess liquid was removed. Direct ^{13}C spectra were acquired using an anti-ring sequence with adiabatic inversion and ^{1}H decoupling with 1024 scans. Solution state experiments were acquired at a magnetic field corresponding to 1200 MHz ^{1}H Larmor frequency at 278 K using a TCI cryoprobe. Data was analyzed in CcpNmr Analysis.v3[6] through NMRBox.[8]

MD Simulations

Input ligand pdb files of PMA were prepared in ChemDraw 3D. For PKC-δ-C1B bound PMA, the PKC-δ-C1B structure was prepared from the PDB entry: 1PTR and protons were added using PyMOL.[10] The phorbol-13-acetate ligand was removed and the PMA ligand was docked onto the PKC-δ-C1B structure using the HADDOCK web server.[11,12] These structures were used in the Membrane Builder module of the CHARMM-GUI online interface[13], which creates a membrane around the inserted ligand using the replacement method. A heterogeneous membrane bilayer was constructed with the lipid compositions shown in SI-2. A water height of 30 was used with the TIP3P water model, and a neutralizing number of ions were added using the replacement method. Input files for GROMACS[14] were generated for use with the CHARMM36m[15] forcefield and a system temperature of 310.15 K. Force field parameterization of PMA was generated using CGenFF.

The membrane systems generated with CHARMM-GUI were energy minimized, equilibrated, and simulated using the CHARMM36m forcefield with GROMACS. Energy minimization was carried out using the steepest descent method for over 5000 steps until a F_{max} <1000 kJ/mol/nm^2 was reached (shown in SI-10). Systems were equilibrated with a pressure of 1 bar using a semi-isotropic Berendsen barostat and reference temperature of 310.15 K using the v-rescale thermostat. All bonds with rigid hydrogen atoms were kept with the LINCS algorithm, and long-range electrostatic interactions were investigated with the particle-mesh Ewald algorithm. Equilibration was assessed by convergence of the area occupied per lipid as shown in SI-10. To generate three independent systems for simulation, the system generated from CHARMM-GUI was equilibrated three independent times and each used as a starting point for three independent production runs. Equilibration was completed over 6 x 500 ps steps. The production runs were carried out at 310.15 K using the Nose-Hoover thermostat and semi-isotropic Parrinello-Rahman barostat with times steps of 2 fs. All systems were simulated out to 500 ns. The last 400 ns were subsequently used in analysis to ensure system stability.

MD Analysis

Structures were extracted and analyzed from complete trajectories using PyMOL software.[10] RMSD values were calculated in PyMOL using the alignment plugin over 5 cycles with outlier rejection. Data was plotted using GraphPad Prism software.

RMSDs for structural ensembles were calculated as an average of RMSDs calculated for structures sampled every 10 ns against the first frame of the simulation. Only the core atoms of the phorbol pharmacophore were used in the RMSD calculation (see Figure 2 of the main article).

The average RMSD for unbound PMA and PKC-bound PMA was calculated for 16 unbound PMA structures extracted every 25 ns from the unbound simulation using a bound structure as reference for the RMSD calculation. RMSDs were then averaged from 10 separate calculations with 10 different bound reference structures spanning the full conformational space of the core pharmacophore.

Supporting Information 2: Membrane compositions used in MD simulations.

PMA bound to PKC-δ-C1B in heterogeneous membranes

**Lipid**	**Upper Leaflet (mole%)**	**Lower Leaflet (mole%)**
PKC-PMA complex	0	1
Cholesterol	14 (21.2%)	13 (24.5%)
Phosphatidylcholine (PC)	13 (19.7%)	12 (22%)
Phosphatidylethanolamine (PE)	18 (27.3%)	17 (32%)
Phosphatidylserine (PS)	8 (12.1%)	7 (13%)
Phosphatidic Acid (PA)	2 (3%)	1 (2%)
Phosphatidylinositol phosphate (PIP)	4 (6.1%)	3 (5.6%)
Ceramide (CER)	7 (10.6%)	6 (10.2%)
Total	66	59

PMA unbound in heterogeneous membranes

**Lipid**	**Upper Leaflet (mole%)**	**Lower Leaflet (mole%)**
PMA	2 (0.0%)	2 (2.2%)
Cholesterol	69 (33.2%)	49 (27.5%)
Phosphatidylcholine (PC)	56 (26.9%)	56 (31.5%)
Phosphatidylethanolamine (PE)	30 (14.4%)	30 (16.9%)
Phosphatidylserine (PS)	0 (0.0%)	20 (11.2%)
Phosphatidic Acid (PA)	0 (0.0%)	2 (1.1%)
Phosphatidylinositol monophosphate (PIP)	0 (0.0%)	8 (4.5%)
Phosphotidylinositol triphosphate (PIP3)	0 (0.0%)	2 (1.1%)
Sphingomyelin (SM)	45 (21.6%)	11 (6.2%)
Galactosyl Ceramide (GlcCer)	6 (2.8%)	0 (0.0%)
Total	208	178

Supporting Information 3: Synthesis of ^{13}C-21,^{13}C-22-PMA:
Preparation of TBS-phorbol 5.25:

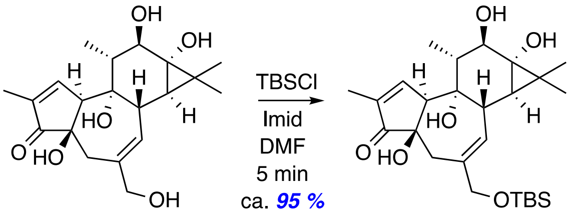

To a flame-dried 20-mL roundbottom flask charged with a stir bar was added phorbol•methanol solvate (phorbol content by qNMR: 170 mg, 0.467 mmol, 1 equiv.; methanol content by qNMR: 30.0 mg, 0.933 mmol, 2 equiv.) followed by the addition of dry DMF (1.0 mL, phorbol concentration: ca. 0.5 M). Imidazole (222.3 mg, 3.3 mmol, 7 equiv.) was added. The reaction mixture was sonicated under Ar atmosphere to ensure homogeneity. The resulting homogenous solution was cooled down to 0 °C, and added TBSCl (232.0 mg, 1.54 mmol, 3.3 equiv.) (**CAUTION**: we noticed on large scale TBSCl addition generated a lot of heat). The reaction flask was removed from the ice bath. TLC was immediately taken, upon which time phorbol was fully consumed. The reaction time was estimated to be ca. 5 minutes. The reaction was quenched by the addition of saturated aqueous NH_4Cl solution (20 mL), followed by extraction with ethyl acetate (5*20 mL or until the organic layer indicated no more product). The combined organic layer was washed with brine (50 mL),[a] dried over Na_2SO_4, filtered, and concentrated. The resulting residue was chromatographed on silica gel with the gradient 50-70 % ethyl acetate/pentane. Product purity was determined by one spot by TLC. Characterization data matched that of previously reported procedure.[16]

[a] The brine wash was crucial for removal of DMF residues.

Preparation of double-labeled 13-($^{13}C_2$)acetate-20-TBS-phorbol 5.35

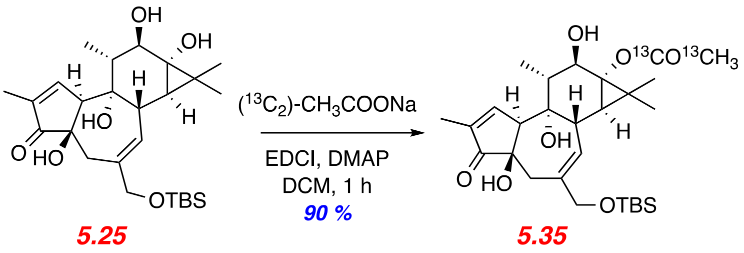

To a vial charged with a stir bar and **5.25** (26.0 mg, 0.054 mmol, 1.0 equiv.) was added sodium $^{13}C_2$-acetate (3.5 mg, 0.042 mmol) and 0.5 mL of THF, followed by the addition of EDCI (26.8 mg, 0.14 mmol, 2.5 equiv.) and DMAP (2.0 mg, 0.0162 mmol, 0.3 equiv.). The reaction mixture was stirred for two hours, during which time the reaction were carefully monitored by TLC. After indication of completion by TLC, the reaction was quenched by 1 mL of saturated Na_2CO_3 aqueous solution. The organic component was separated, and the aqueous layer was washed with ethyl acetate (2 mL×5 times). The combined organic layer was dried over Na_2SO_4, concentrated *in vacuo*, and the residue was chromatographed with the eluent EtOAc/pentane = 1:1 to afford the desired product as a white solid (25.4 mg, 90 %). Product purity was determined as one spot by TLC.

TLC: R_f 0.5 (50 % EtOAc/pentane, UV active, green by *p*-anisaldehyde stain)

^{1}H-NMR ($CDCl_3$, 600 MHz): δ 7.58 (s, 1H), 5.61 (d, J = 4.7 Hz), 4.03–3.99 (m, 3H), 3.15–3.14 (m, 2H), 2.47 (d, 1H, J = 19 Hz), 2.37 (d, 1H, J = 19 Hz), 2.18 (br s, 1H), 2.12 (dd, 3H, J_1 = 130 Hz, J_2 = 6.8 Hz), 2.00 (dq, 1H, J_1 = 6.5 Hz, J_2 = 9.7 Hz), 1.26 (s, 3H), 1.22 (s, 3H), 1.09 (d, 3H, J = 6.5 Hz), 1.03 (d, 1H, J = 5.6 Hz), 0.89 (s, 9H), 0.061 (s, 3H), and 0.055 (s, 3H) ppm.

^{13}C-NMR ($CDCl_3$, 125 MHz): δ 209.0, 174.4 (d, J = 58.9 Hz), 160.5, 140.7, 133.1, 127.7, 78.4, 77.6, 73.7, 68.0, 57.0, 45.2, 39.2, 38.5, 35.5, 29.9, 26.1, 23.8, 21.2 (d, J = 58.9), 18.5, 17.0, 15.3, 10.3, and -5.1 ppm.

$[\alpha]^{23.3}_D$ = +14.5 ° (c = 0.150, CH_2Cl_2).

IR: 3421, 2954, 2927, 2884, 2856, 1743, 1694, 1682, 1629, 1471, 1462, 1424, 1390, 1377, 1360, 1327, 1251, 1225, 1187, 1135, 1082, 1062, 1025, 1007, 988, and 940 cm^{-1}.

HRMS (ESI-TOF): Calcd for $C_{26}{}^{13}C_2H_{44}O_7SiNa^+$ $[M+Na]^+$ requires 545.2822, found 545.2816.

^{1}H-NMR ($CDCl_3$, 600 MHz):

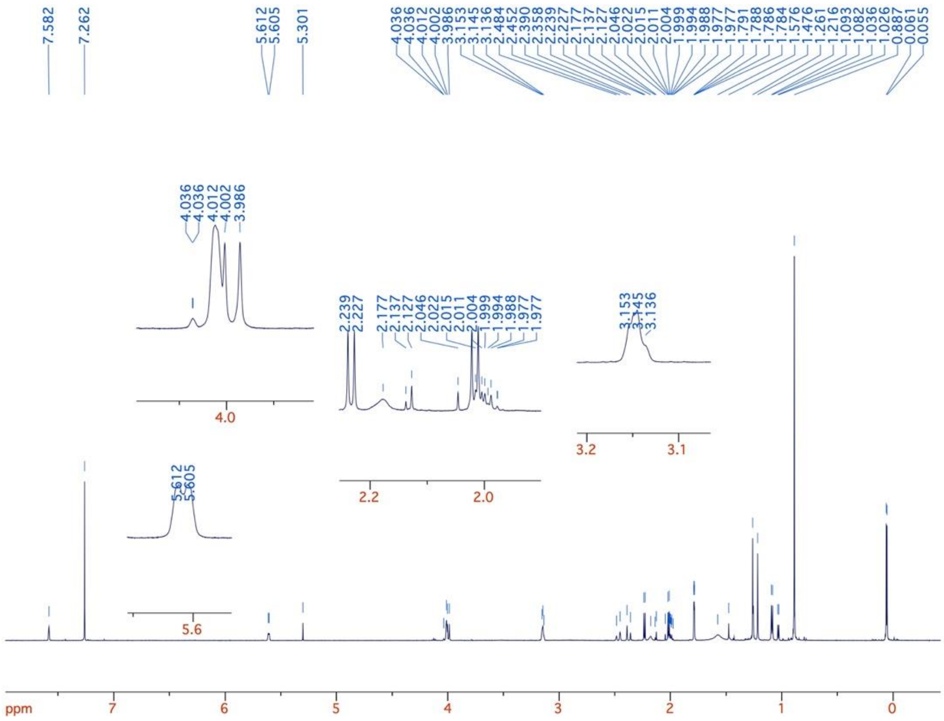

^{13}C-NMR ($CDCl_3$, 125 MHz):

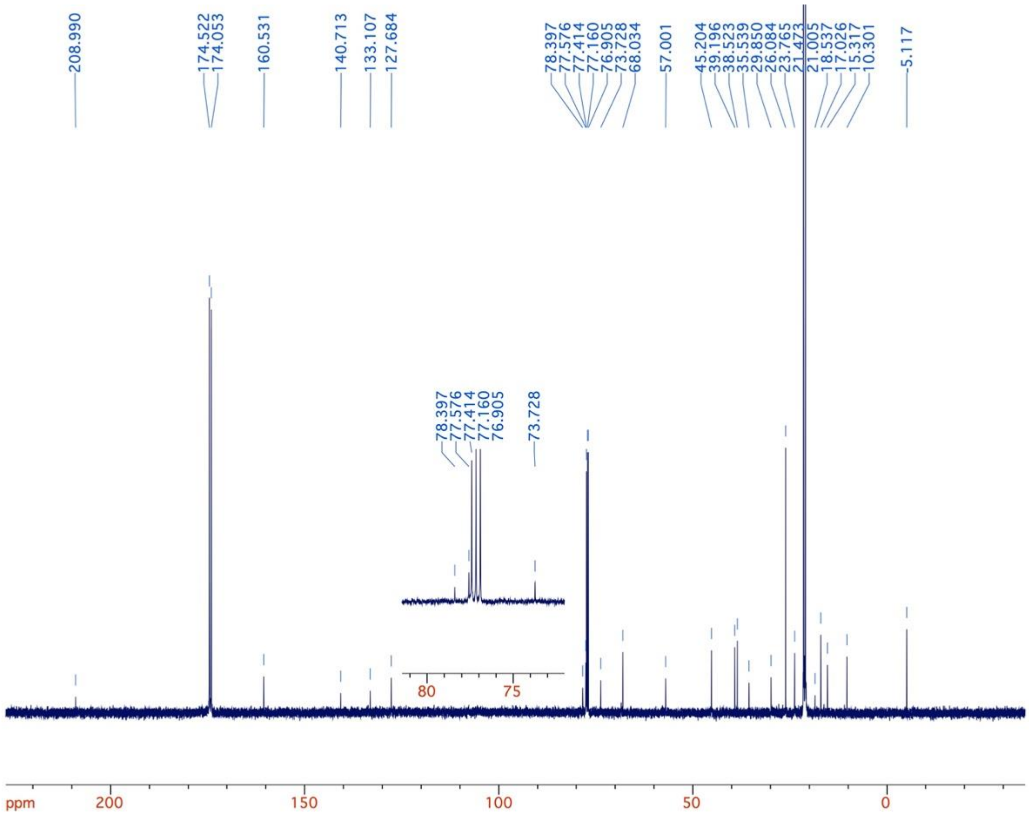

Preparation of phorbol 13-($^{13}C_2$)-acetate-12-myristate (5.34):

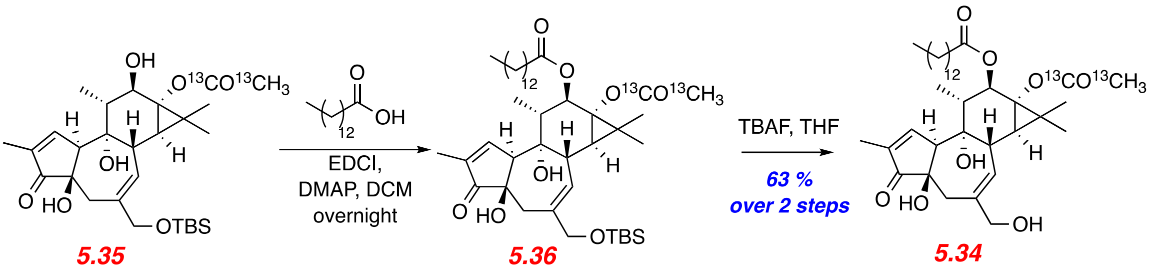

To a vial containing **5.35** (17.5 mg, 0.034 mmol, 1.0 equiv.) was added 1.5 mL of DCM, followed by the addition of myristic acid (76.7 mg, 0.34 mmol, 10 equiv.), ECDI (52.8 mg, 0.34 mmol, 10 equiv.), and DMAP (4.1 mg, 0.034 mmol, 1.0 equiv.). The reaction mixture was stirred overnight, upon which time TLC indicated complete conversion. The reaction mixture was quenched by saturated $NaHCO_3$ aqueous solution (10 mL), followed by addition of 10 mL of ethyl acetate. The organic layer was separated, and the aqueous layer was washed with ethyl acetate (10 mL×5 times). The combined organic component was dried over Na_2SO_4, concentrated *in vacuo*, and the residue was passed through a plug of silica gel with a 9:1 pentane/EtOAc eluent to yield the 20-(*tert*-butyldimethylsilyl)-PMA **5.36** contaminated with a small amount of myristic acid. This material could be subjected to the next step without further purification.

The crude product **5.36** was dissolved in 1.5 mL of THF. The solution was cooled to 0 ˚C, followed by the addition of 0.07 mL of 1 M TBAF in THF (0.07 mmol, 2.0 equiv.). The reaction mixture was stirred for 30 minutes, during which time the color was changing from colorless, clear to light orange. After TLC indicated full conversion, the reaction was quenched by saturated NH_4Cl aqueous solution, followed by the addition of 10 mL of ethyl acetate. The organic layer was separated, and the aqueous layer was washed with ethyl acetate (10 mL×5 times). The combined organic component was dried over Na_2SO_4, concentrated *in vacuo*, and the residue was chromatographed with EtOAc/pentane = 1:1 as the eluent to yield the desired product as a while solid (13 mg, 63.7 %). Product purity was determined as one spot by TLC.

TLC: R_f 0.5 (50 % EtOAc/pentane, UV active, black by *p*-anisaldehyde stain)

^{1}H-NMR ($CDCl_3$, 600 MHz): δ 7.59 (s, 1H), 5.68 (d, 1H, J = 5.4 Hz), 5.54 (brs, 1H), 5.41 (d, 1H, J = 10.3 Hz), 4.04 (d, 1H, J = 13.1 Hz), 4.00 (d, 1H, J = 13 Hz), 3.25–3.22 (m, 2H), 2.54 (d, 1H, 19.0 Hz), 2.48 (d, 1H, 19.1 Hz), 2.37–2.28 (m, 2H), 2.13 (dq, 1H, J_1 = 6.2 Hz, J_2 = 10.0 Hz), 2.09 (dd, 3H, J_1 = 130 Hz, J_2 = 6.8 Hz), 1.78 (dd, 3H, J_1 = 1.2 Hz, J_2 = 2.8 Hz), 1.63 (quintet, 2H, J = 7.2 Hz), 1.25 (m, 23H), 1.24 (s, 3H), 1.21 (s, 3H), 1.08 (d, 1H, J = 5.2 Hz), and 0.90–0.87 (m, 6H).

^{13}C-NMR ($CDCl_3$, 125 MHz): δ 209.0, 173.9 (d, J = 59 Hz), 160.9, 140.5, 133.0, 129.3, 78.3, 76.6, 73.8, 68.2, 56.3, 43.0, 39.2, 38.7, 36.3, 34.7, 32.1, 29.82, 29.79, 29.7, 29.74, 29.66, 29.5, 29.4, 29.2, 25.3, 24.0, 22.8, 21.3, 21.2 (d, J = 59.2 Hz), 21.0, 16.9, 14.6, 14.3, and 10.3.

IR: 3420, 2924, 2854, 1736, 1705, 1682, 1629, 1461, 1375, 1356, 1325, 1249, 1223, 1178, 1109, 1078, 1061, 987, 946, 913, 886, and 863 cm^{-1}.

$[\alpha]^{24.0}_D$ = +42.0 ° (c = 0.200, CH_2Cl_2).

HRMS: (ESI-TOF) Calcd for $C_{34}{}^{13}C_2H_{56}O_8Na^+$ $[M+Na]^+$ requires 641.3941, found 641.3934.

^{1}**H-NMR** ($CDCl_3$, 600 MHz):

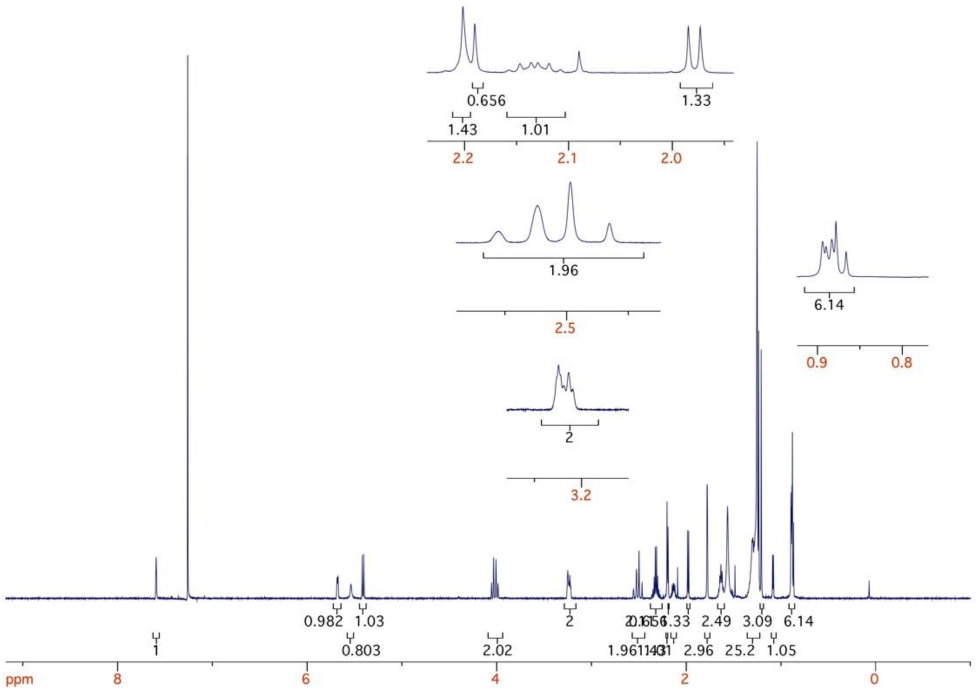

^{13}**C-NMR** ($CDCl_3$, 125 MHz):

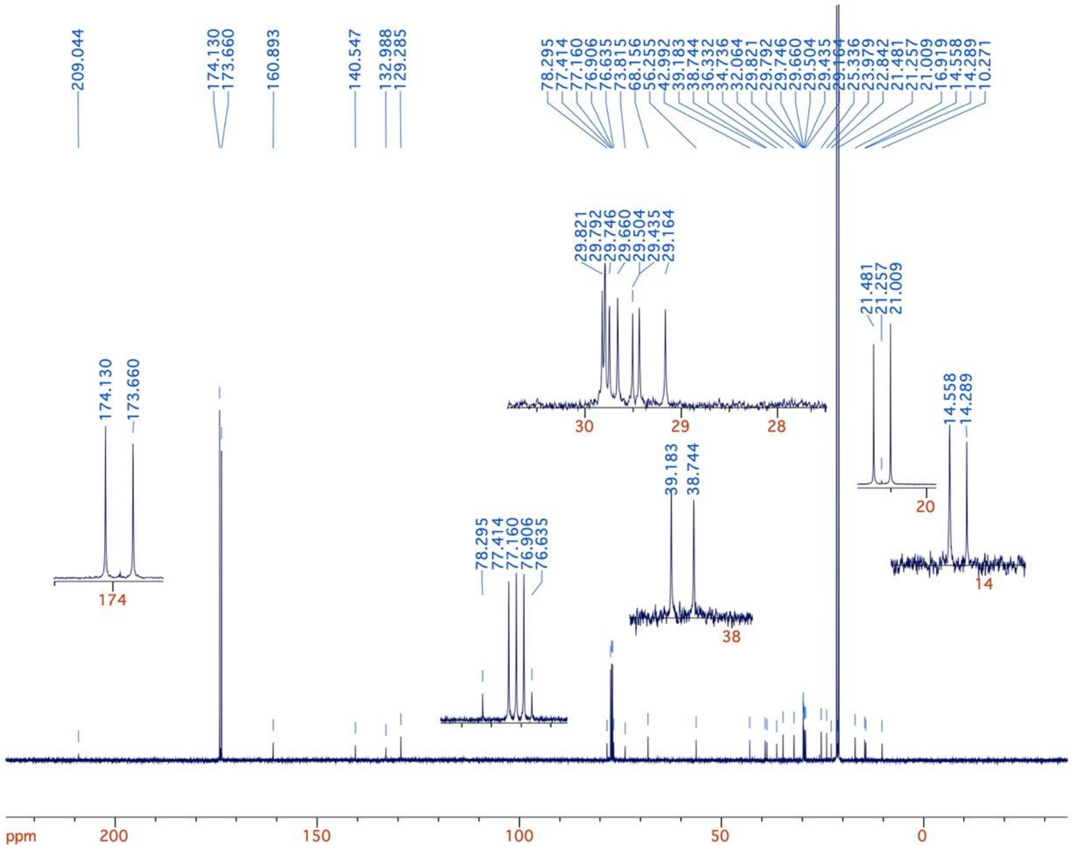

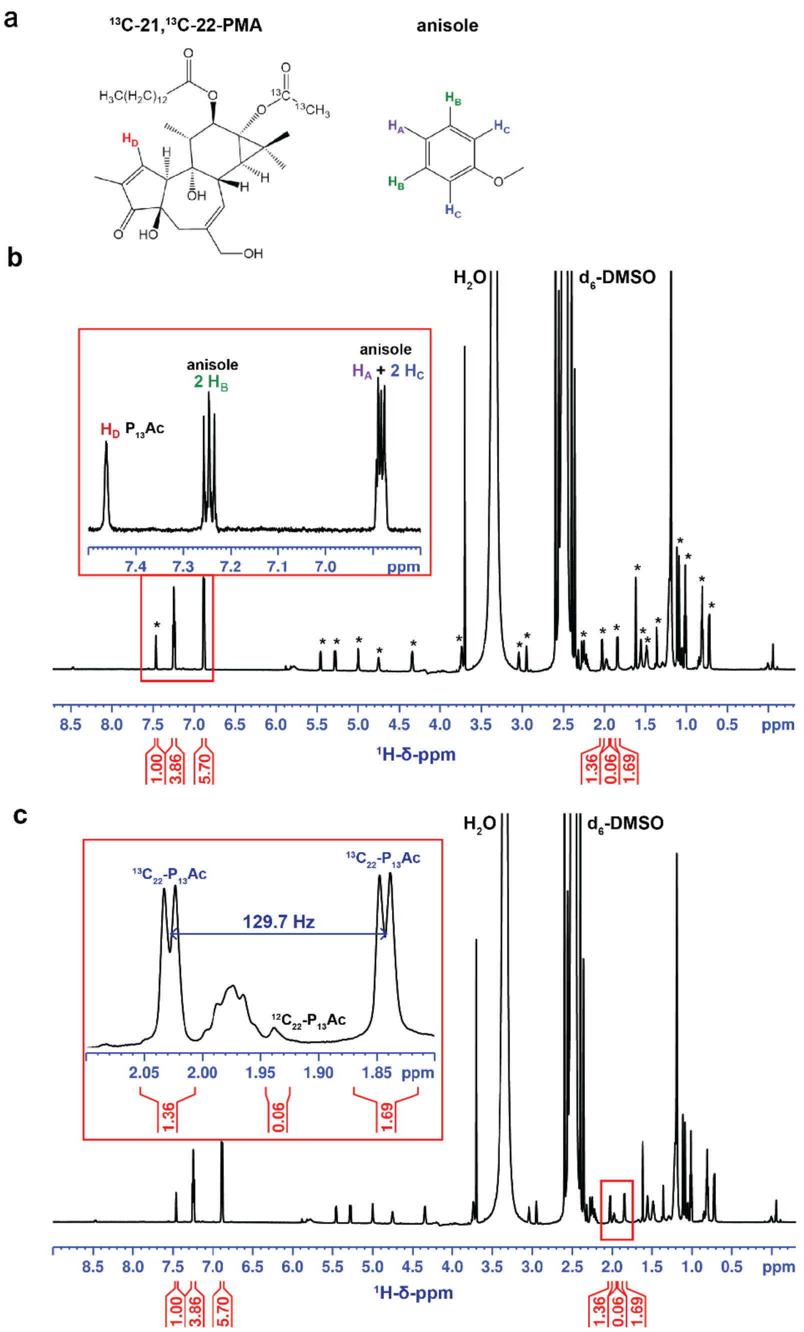

Supporting Information 4: Measurement of ^{13}C-21,^{13}C-22-PMA concentration by NMR. a) Chemical structure of PMA and anisole with the ^{1}H atoms used in the analysis highlighted. b) PMA concentration determined by comparison to anisole standard. PMA was diluted 1/100 in DMSO and anisole was added to 400 µM. The inset shows the distinct $^{1}H_{1}$ peak (H_D) peak of PMA at 7.46 ppm and the aromatic peaks of anisole peaks at (H_A and H_C) 7.25 ppm and (H_B) 6.89 ppm respectively. The * indicates PMA peaks. The integrals of each peak are shown below the axis in red. Comparing the integral we obtain a PMA concentration that is 200 µM (after 1/100 dilution). Giving an undiluted stock concentration of 20 mM. The data was collected with 32 scans. c) Analysis of PMA ^{13}C labeling. The inset shows the doublet corresponding to the ^{13}C-22 atom of PMA with a small singlet halfway between corresponding to unlabeled ^{12}C-22 atom of PMA. Peak integration indicates that at least 98% of the ^{13}C-22 atoms are fully labeled. Data was acquired with 3328 scans. All experiments were conducted at 700 MHz ^{1}H Larmor frequency at 298 K).

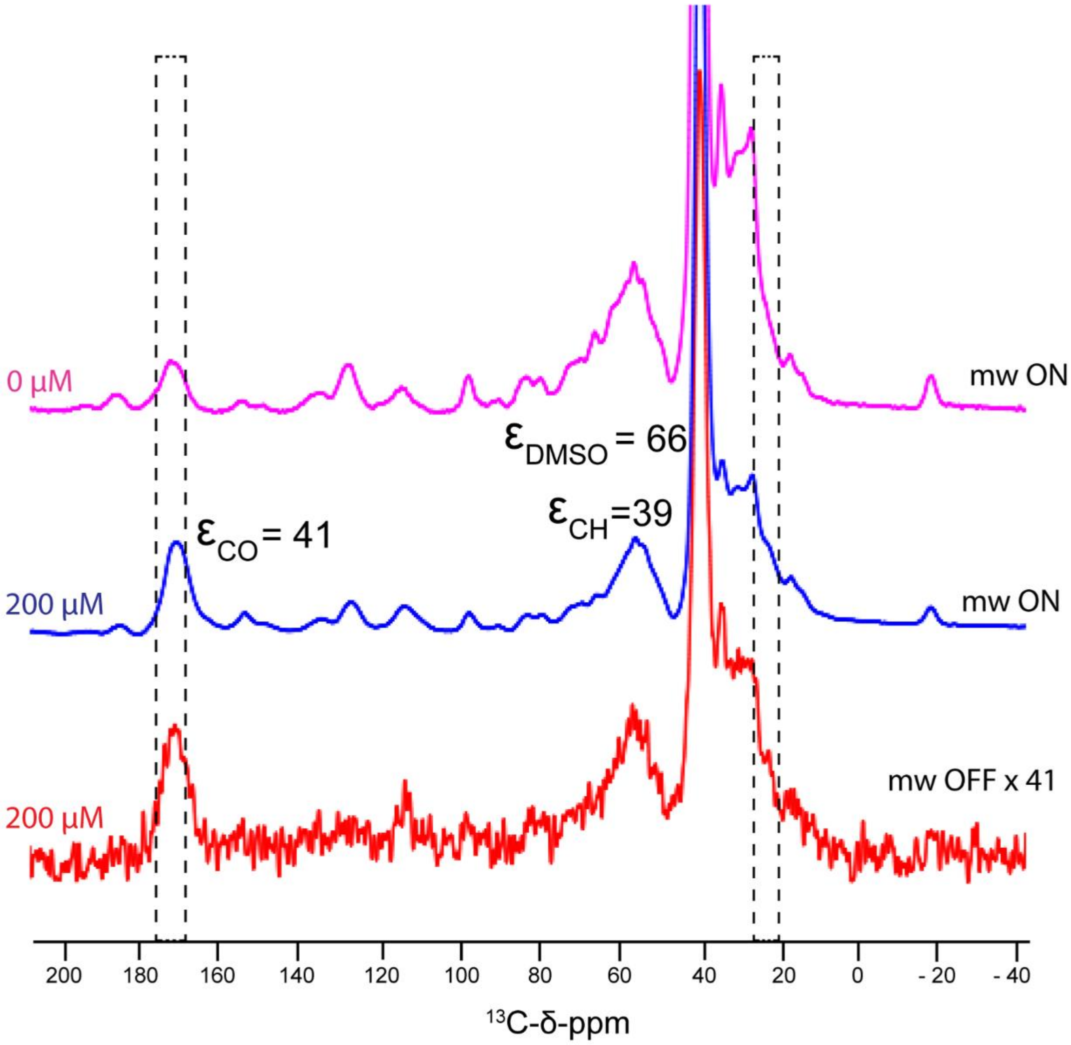

Supporting Information 5: DNP enhancements of JLat 9.2 T cells with 5 mM AsymPolPOK
Enhancement of natural abundance ^{13}C in JLat 9.2 T cells. The dashed boxes indicate the expected chemical shift position of the labeled ^{13}C-21,^{13}C-22-PMA. Spectra acquired with microwaves are shown in blue and without microwaves shown in red. The enhancement is determined by the ratio of the signal intensity between these two spectra. The spectra shown in magenta is the microwave on spectrum of JLat 9.2 T cells with no ^{13}C-21,^{13}C-22-PMA.

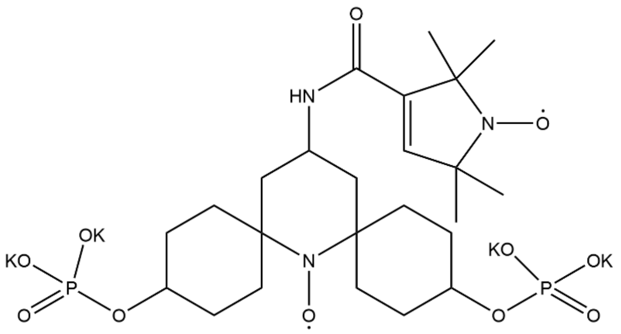

Supporting Information 6: Structure of AsymPolPOK. Structure of the polarizing agent used for in-cell DNP-NMR.

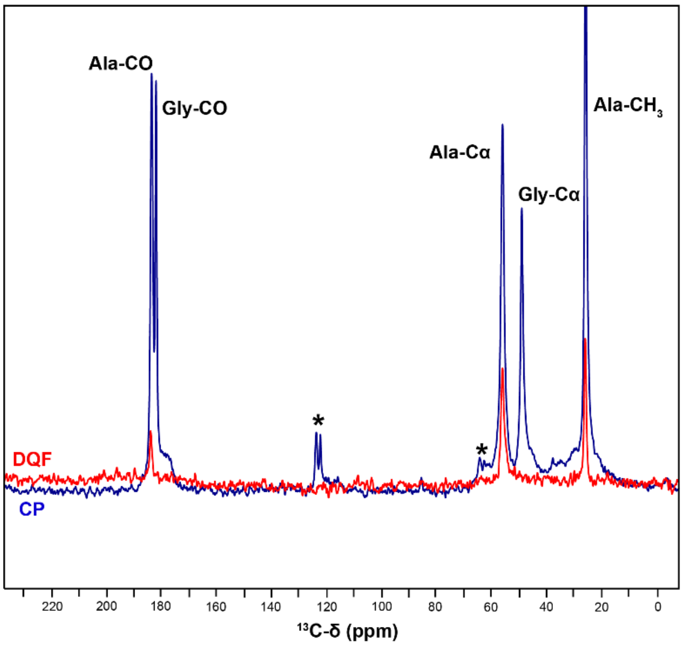

	Ala-CO	Ala-Cα	Ala-CH₃
Transfer efficiency compared to CP	11.9 %	20.8 %	35.9 %

Supporting Information 7: Efficiency of SPC5 double quantum filter with U-^{13}C-Alanine
Transfer efficiency of SPC5 Double Quantum Filtered (DQF) compared to cross-polarization (CP) of a mixture of powdered U-^{13}C-Alanine and NA-Glycine acquired at 600 MHz ^{1}H Larmor frequency, 9 kHz MAS at 275 K and the carrier frequency at 170 ppm (optimized for CO intensity). * indicates spinning side bands. The efficiency is a comparative measure of the signal intensity of peaks acquired with the SPC-DQF compared to the signal intensity achieved using CP.

2.0 mM ^{13}C-21,22-PAc

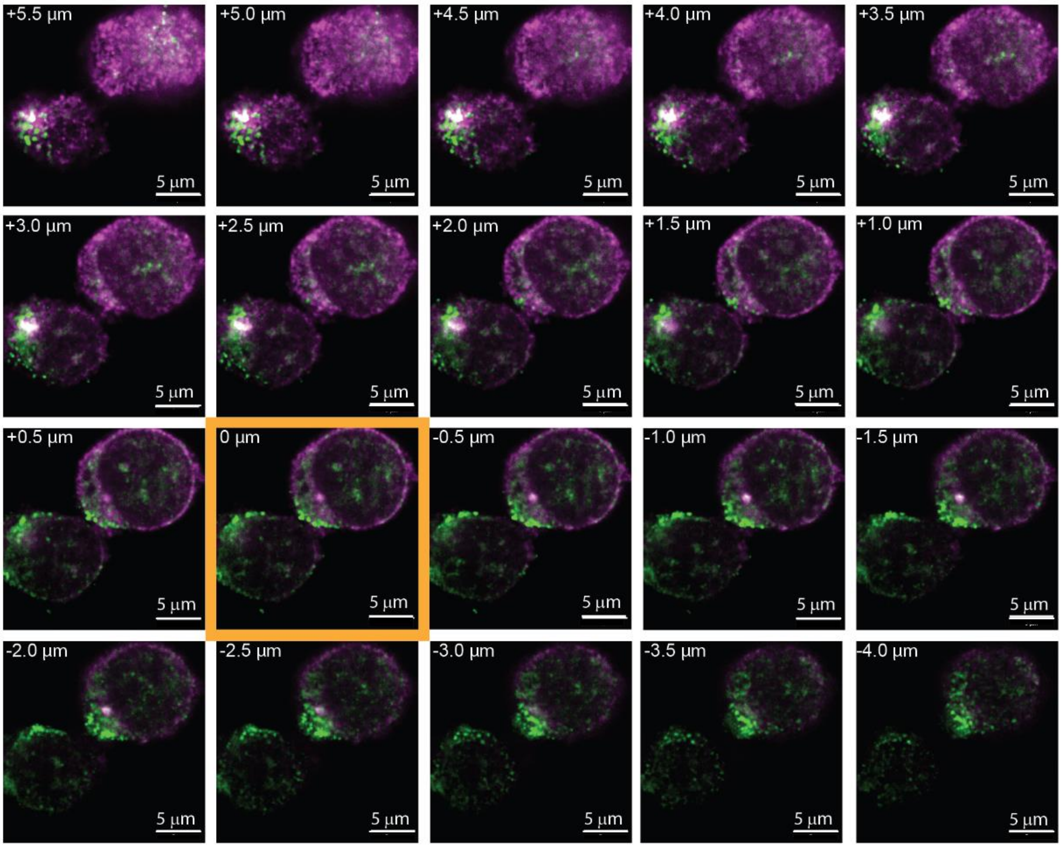

200.0 µM ^{13}C-21,22-PAc

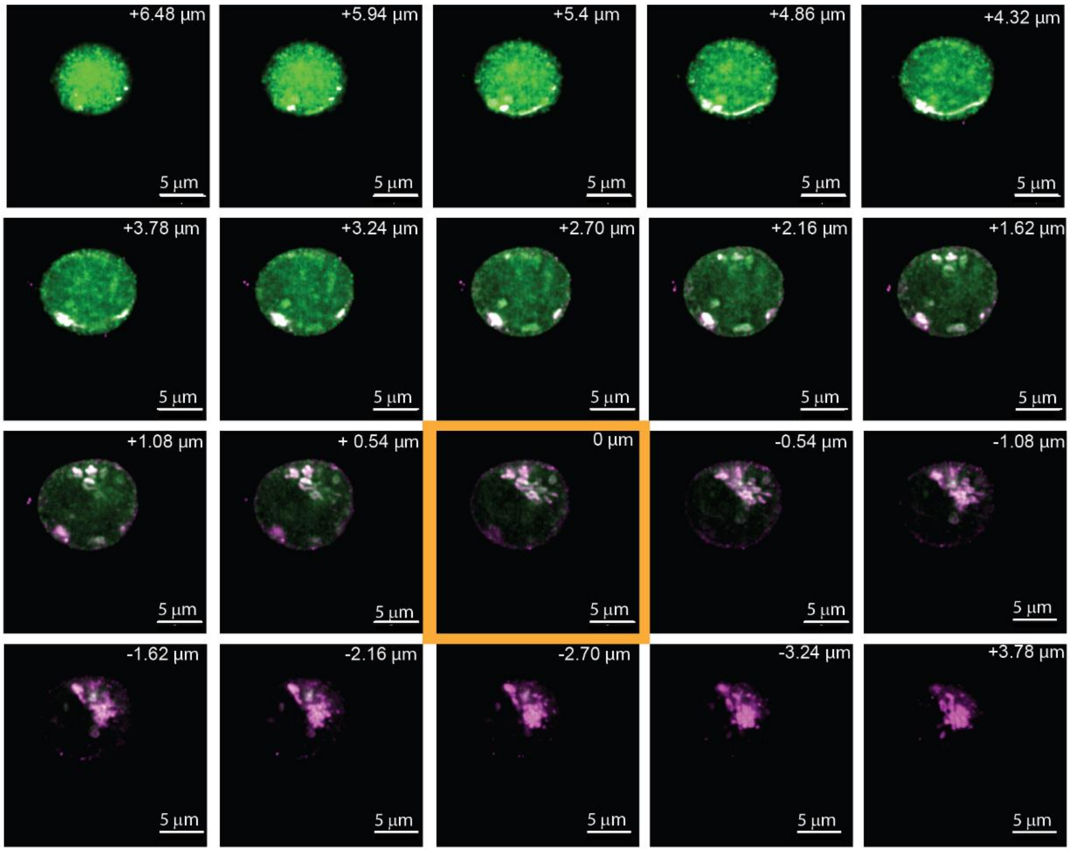

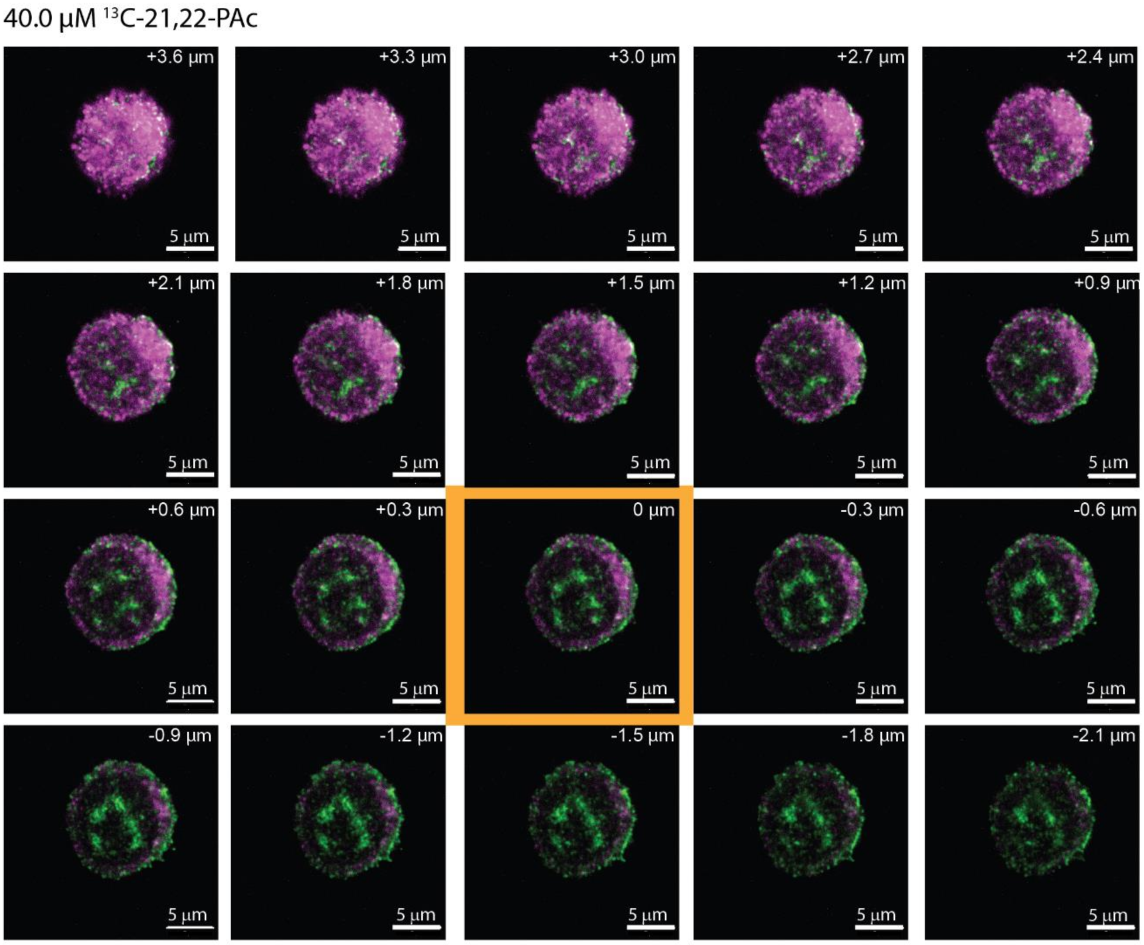

Supporting Information 8: Image series of PKC-δ translocation with ^{13}C-21,^{13}C-22-PMA. z-stack series of each sample analyzed in Figure 3 starting from the bottom of the cell. Shown are overlays of PKC-δ (magenta) and Flotillin-1 (green). The images shown in Figure 3 are taken from this series and are highlighted in orange. Cells were imaged using a Nikon Ti2 microscope equipped with a confocal re-scan module. The z-position relative to the highlighted image is given in the top right corner of each image in µm. The images of the control 0 µm treated cells were not collected as a z-stack and thus the complete image series is shown in Figure 3 of the main article.

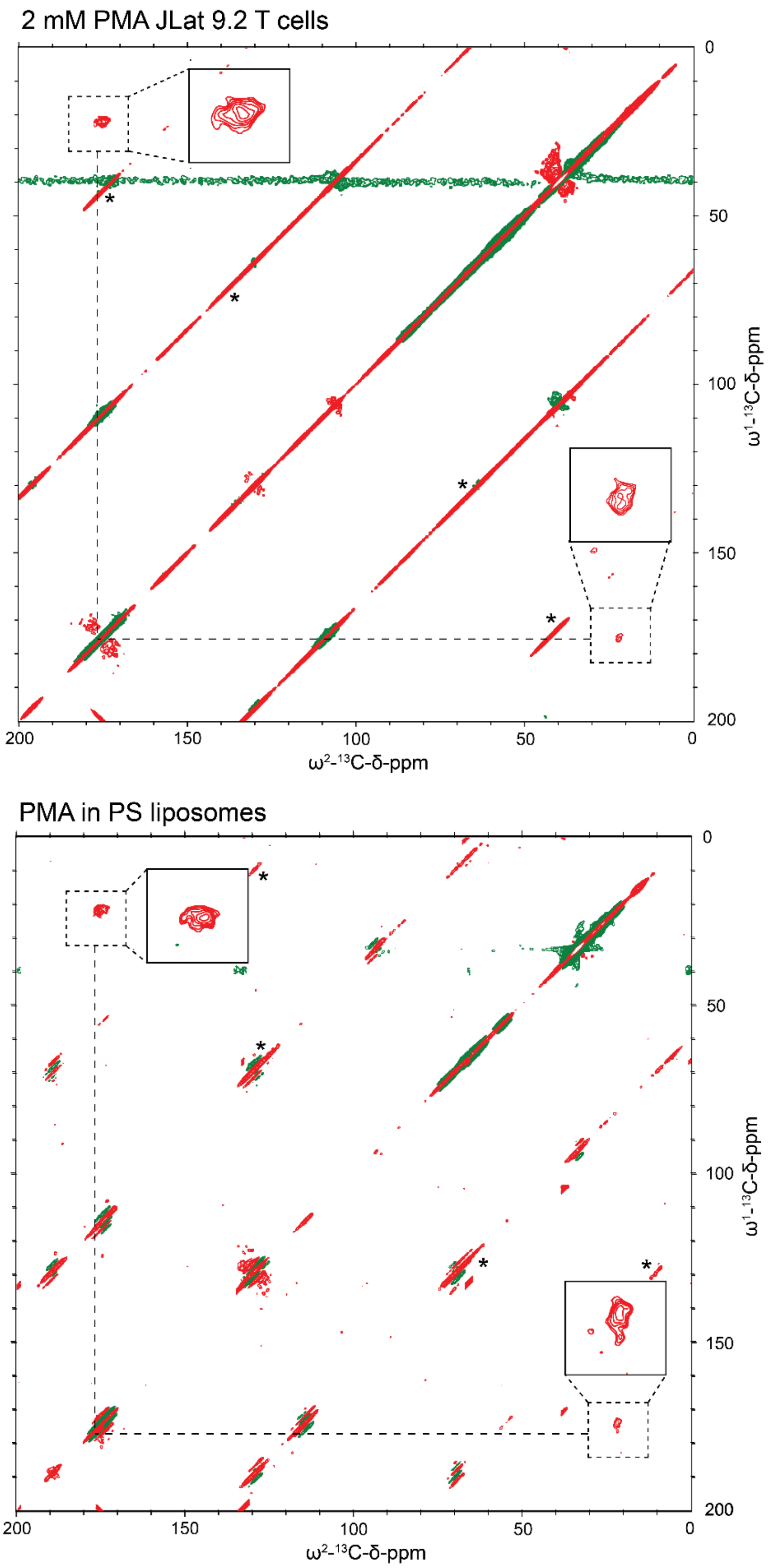

Supporting Information 9: 2D-DARR spectrum of ^{13}C-21,^{13}C-22-PMA in JLat 9.2 T cells

DARR spectrum showing cross peaks selective for ^{13}C-21,^{13}C-22-PMA (indicated by the dashed boxes). * indicate spinning side bands. Diagonal peaks arise from natural abundance signals. Data was collected at 600 MHz Larmor frequency, 9 kHz magic angle spinning and 20 ms DARR mixing at 114 K.

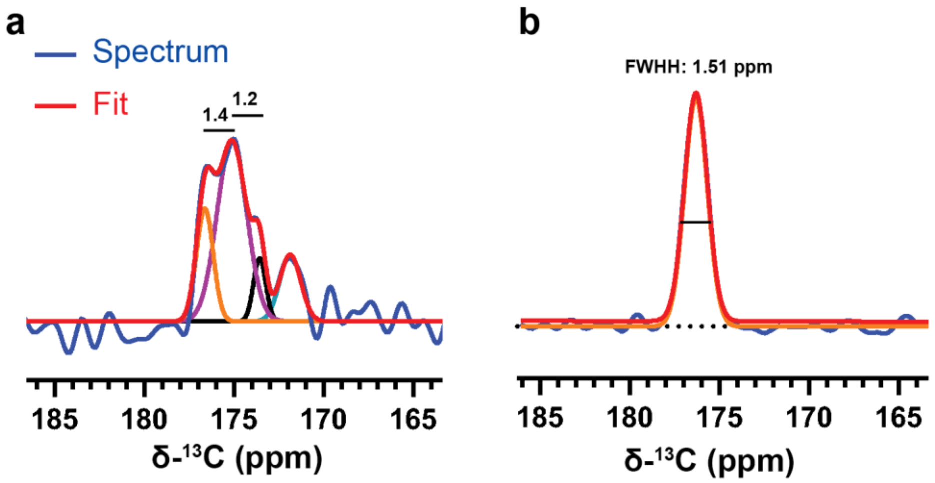

Supporting Information 10: Linewidth analysis of DARR cross peaks.
1D slice from the DARR spectrum through the carbonyl-methyl cross peak. Peaks were fit to a gaussian lineshape using DMFit to a) 4 peaks in 200 µM JLat 9.2 T cell spectra or b) 1 peak in PS liposomes with PKC-δC1B spectra to a standard deviation of < 3.0.

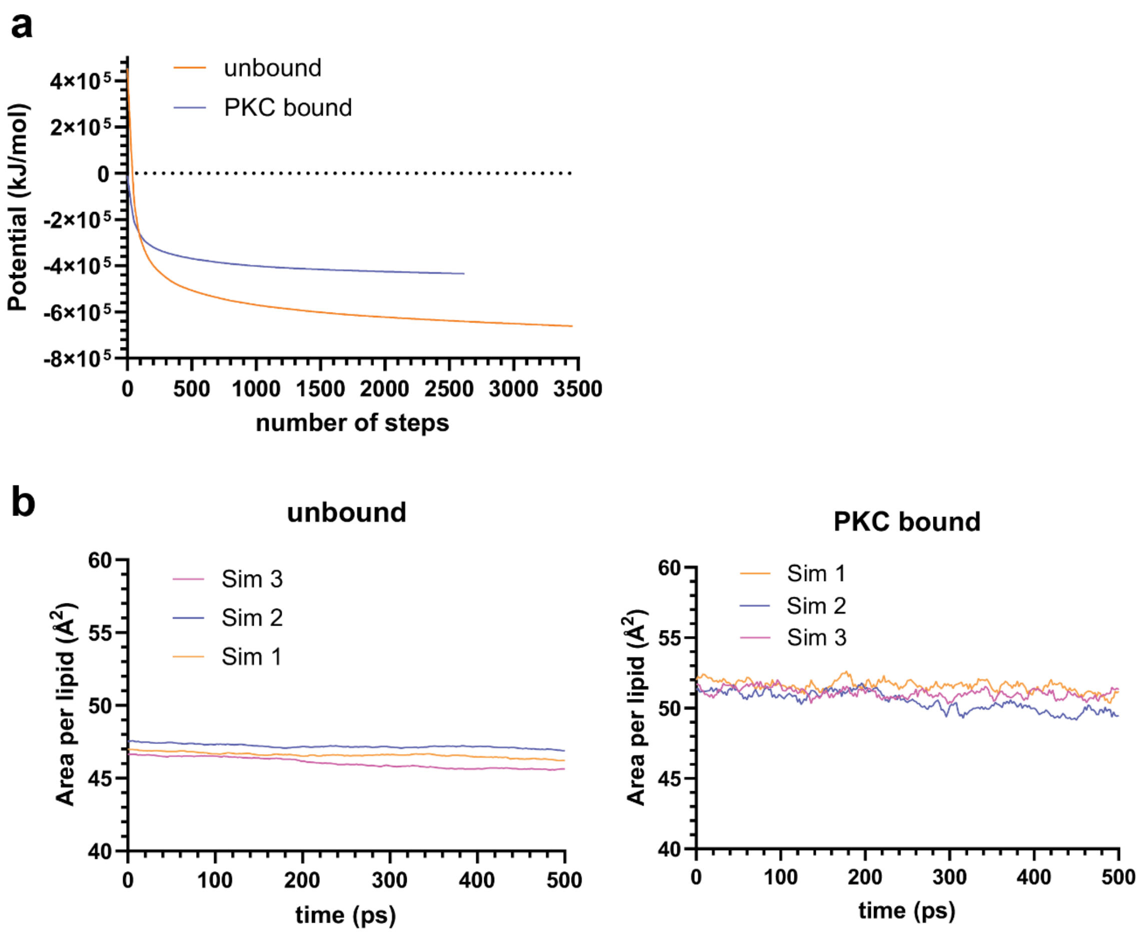

Supporting Information 11: Minimization and equilibration of membrane systems in GROMACs. a) Energy minimization by steepest descent method. Minimization was carried out over a maximum of 5000 steps or until a Fmax <1000 kJ/mol was reached. Convergence of the potential energy of the system is shown. b) Equilibration of membrane systems was assessed by stabilization of the area per lipid over the final equilibration period (500 ps). Total equilibration period was 3000 ps.

References

(1) Jordan, A.; Defechereux, P.; Verdin, E. The Site of HIV-1 Integration in the Human Genome Determines Basal Transcriptional Activity and Response to Tat Transactivation. *EMBO J* **2001**, *20* (7), 1726–1738. https://doi.org/10.1093/emboj/20.7.1726.

(2) Mentink-Vigier, F.; Marin-Montesinos, I.; Jagtap, A. P.; Halbritter, T.; van Tol, J.; Hediger, S.; Lee, D.; Sigurdsson, S. T.; De Paëpe, G. Computationally Assisted Design of Polarizing Agents for Dynamic Nuclear Polarization Enhanced NMR: The AsymPol Family. *J Am Chem Soc* **2018**, *140* (35), 11013–11019. https://doi.org/10.1021/jacs.8b04911.

(3) Overall, S. A.; Price, L. E.; Albert, B. J.; Gao, C.; Alaniva, N.; Judge, P. T.; Sesti, E. L.; Wender, P. A.; Kyei, G. B.; Barnes, A. B. In Situ Detection of Endogenous HIV Activation by Dynamic Nuclear Polarization NMR and Flow Cytometry. *Int J Mol Sci* **2020**, *21* (13), 4649. https://doi.org/10.3390/ijms21134649.

(4) Overall, S. A.; Barnes, A. B. Biomolecular Perturbations in In-Cell Dynamic Nuclear Polarization Experiments. *Front Mol Biosci* **2021**, *8*, 743829. https://doi.org/10.3389/fmolb.2021.743829.

(5) Hohwy, M.; Rienstra, C. M.; Jaroniec, C. P.; Griffin, R. G. Fivefold Symmetric Homonuclear Dipolar Recoupling in Rotating Solids: Application to Double Quantum Spectroscopy. *J Chem Phys* **1999**, *110* (16), 7983–7992. https://doi.org/10.1063/1.478702.

(6) Skinner, S. P.; Fogh, R. H.; Boucher, W.; Ragan, T. J.; Mureddu, L. G.; Vuister, G. W. CcpNmr AnalysisAssign: A Flexible Platform for Integrated NMR Analysis. *J Biomol NMR* **2016**, *66* (2), 111–124. https://doi.org/10.1007/s10858-016-0060-y.

(7) Lee, W.; Tonelli, M.; Markley, J. L. NMRFAM-SPARKY: Enhanced Software for Biomolecular NMR Spectroscopy. *Bioinformatics* **2015**, *31* (8), 1325–1327. https://doi.org/10.1093/bioinformatics/btu830.

(8) Maciejewski, M. W.; Schuyler, A. D.; Gryk, M. R.; Moraru, I. I.; Romero, P. R.; Ulrich, E. L.; Eghbalnia, H. R.; Livny, M.; Delaglio, F.; Hoch, J. C. NMRbox: A Resource for Biomolecular NMR Computation. *Biophys J* **2017**, *112* (8), 1529–1534. https://doi.org/https://doi.org/10.1016/j.bpj.2017.03.011.

(9) Massiot, D.; Fayon, F.; Capron, M.; King, I.; Le Calve, S.; Alonso, B.; Durand, J.-O.; Bujoli, B.; Gan, Z.; Hoatson, G. Modelling One- and Two-Dimensional Solid-State NMR Spectra. *Magn Reson Chem* **2002**, *40* (1), 70–76.

(10) Schrödinger, L. *The {PyMOL} Molecular Graphics System, Version 1.8*; 2015.

(11) van Zundert, G. C. P.; Rodrigues, J. P. G. L. M.; Trellet, M.; Schmitz, C.; Kastritis, P. L.; Karaca, E.; Melquiond, A. S. J.; van Dijk, M.; de Vries, S. J.; Bonvin, A. M. J. J. The HADDOCK2.2 Web Server: User-Friendly Integrative Modeling of Biomolecular Complexes. *J Mol Biol* **2016**, *428* (4), 720–725. https://doi.org/https://doi.org/10.1016/j.jmb.2015.09.014.

(12) Honorato, R. V; Koukos, P. I.; Jiménez-García, B.; Tsaregorodtsev, A.; Verlato, M.; Giachetti, A.; Rosato, A.; Bonvin, A. M. J. J. Structural Biology in the Clouds: The WeNMR-EOSC Ecosystem. *Front Mol Biosci* **2021**, *8*, 729513. https://doi.org/10.3389/fmolb.2021.729513.

(13) Jo, S.; Kim, T.; Iyer, V. G.; Im, W. CHARMM-GUI: A Web-Based Graphical User Interface for CHARMM. *J Comput Chem* **2008**, *29* (11), 1859–1865. https://doi.org/10.1002/JCC.20945.

(14) Brooks, B. R.; Brooks, C. L.; Mackerell, A. D.; Nilsson, L.; Petrella, R. J.; Roux, B.; Won, Y.; Archontis, G.; Bartels, C.; Boresch, S.; Caflisch, A.; Caves, L.; Cui, Q.; Dinner, A. R.; Feig, M.; Fischer, S.; Gao, J.; Hodoscek, M.; Im, W.; Kuczera, K.; Lazaridis, T.; Ma, J.; Ovchinnikov, V.; Paci, E.; Pastor, R. W.; Post, C. B.; Pu, J. Z.; Schaefer, M.; Tidor, B.; Venable, R. M.; Woodcock, H. L.; Wu, X.; Yang, W.; York, D. M.; Karplus, M. CHARMM: The Biomolecular Simulation Program. *J Comput Chem* **2009**, *30* (10), 1545. https://doi.org/10.1002/JCC.21287.

(15) Lee, J.; Cheng, X.; Swails, J. M.; Yeom, M. S.; Eastman, P. K.; Lemkul, J. A.; Wei, S.; Buckner, J.; Jeong, J. C.; Qi, Y.; Jo, S.; Pande, V. S.; Case, D. A.; Brooks, C. L.; MacKerell, A. D.; Klauda, J. B.; Im, W. CHARMM-GUI Input Generator for NAMD, GROMACS, AMBER, OpenMM, and CHARMM/OpenMM Simulations Using the CHARMM36 Additive Force Field. *J Chem Theory Comput* **2016**, *12* (1), 405–413. https://doi.org/10.1021/ACS.JCTC.5B00935/ASSET/IMAGES/LARGE/CT-2015-00935E_0005.JPEG.

(16) Matsuya, Y.; Yu, Z.; Yamamoto, N.; Mori, M.; Saito, H.; Takeuchi, M.; Ito, M.; Nemoto, H. Synthesis of New Phorbol Derivatives Having Ethereal Side Chain and Evaluation of Their Anti-HIV Activity. *Bioorg Med Chem* **2005**, *13* (14), 4383–4388. https://doi.org/10.1016/j.bmc.2005.04.056.
